# Supplementary material for: A RHO Small GTPase Regulator ABR Secures Mitotic Fidelity in Human Embryonic Stem Cells
Source: Stem Cell Reports. 2017 Jun 1;9(1):58–66. doi: 10.1016/j.stemcr.2017.05.003 (PMC5511046; doi:10.1016/j.stemcr.2017.05.003)
Supplement: Document S1. Supplemental Experimental Procedures and Figures S1–S3 [file mmc1.pdf]

Stem Cell Reports, Volume *9*

Supplemental Information

A RHO Small GTPase Regulator ABR Secures Mitotic Fidelity in Human Embryonic Stem Cells

Masatoshi Ohgushi, Maki Minaguchi, Mototsugu Eiraku, and Yoshiki Sasai

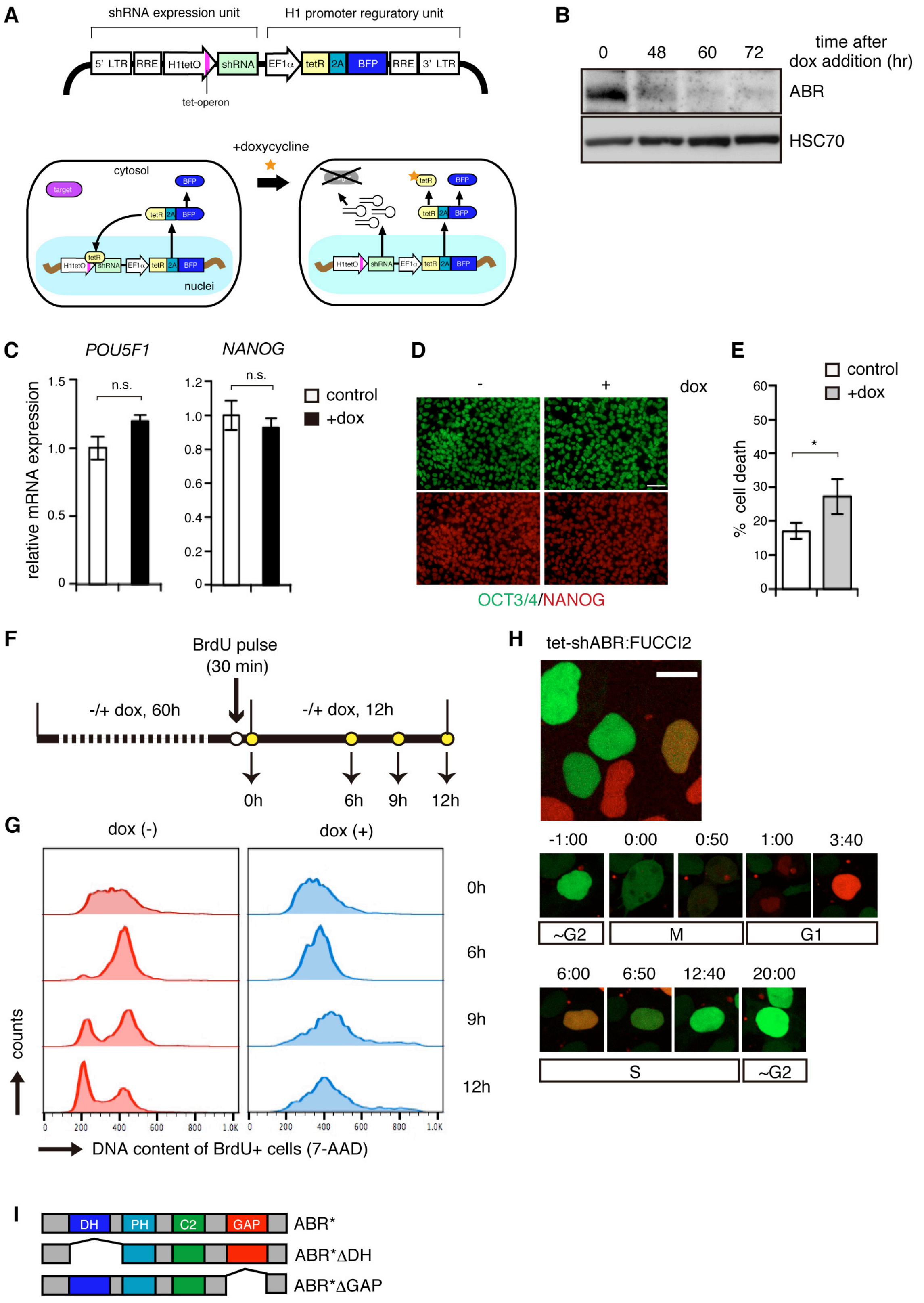
A
shRNA expression unit
H1 promoter regulatory unit
5' LTR
RRE
H1tetO
shRNA
EF1α
tetR
2A
BFP
RRE
3' LTR
tet-operon
+doxycycline
cytosol
target
nuclei
B
time after dox addition (hr)
0
48
60
72
ABR
HSC70
C
POU5F1
NANOG
relative mRNA expression
n.s.
control
+dox
D
dox
OCT3/4/NANOG
E
% cell death
control
+dox
F
BrdU pulse (30 min)
-/+ dox, 60h
-/+ dox, 12h
0h
6h
9h
12h
G
dox (-)
dox (+)
counts
DNA content of BrdU+ cells (7-AAD)
H
tet-shABR:FUCCI2
-1:00
0:00
0:50
1:00
3:40
~G2
M
G1
6:00
6:50
12:40
20:00
S
~G2
I
DH
PH
C2
GAP
ABR*
ABR*ΔDH
ABR*ΔGAP

Figure S1. ABR depletion strategy and phenotypic analyses, related to Figure 1.

(A) Overview of drug-inducible gene silencing strategy. The activity of a modified *H1* promoter was minimized by co-expressing Tet Repressor proteins (TetR) driven from a constitutively active *EF1A* promoter. Doxycyclin supplementation relieves the modified *H1* promoter from TetR, leading to the induction of shRNA transcription. LTR, long terminal repeat; RRE, rev response element; FP, fluorescence protein.

(B) Time course analysis of ABR protein downregulation.

(C-D) The effects of ABR depletion on pluripotent marker expression. Cells were cultured with or without dox for 3 days, and as markers for pluripotency, *POU5F1* and *NANOG* mRNA expression were evaluated by qPCR (C). Data are displayed as relative value to control. The protein abundance of OCT3/4 (green) and NANOG (red) were also evaluated by immunostaining (D). Scale bar represents 50 µm.

(E) Cell death assay. Tet-shABR KhES-1 cells were cultured with or without dox for 3 days. Dead cells were identified by an incorporation of live cell-impermeable DNA dye DRAQ7. DRAQ7-positive dead cells were determined by flow cytometric analyses.

(F-G) Tracing analyses of S-phase labeled cells. (F) A schematic diagram for the time schedule. S-phase cells were labeled by a transient supplementation of BrdU at 60 h time points during dox treatment. Then, cells were harvested at the indicated time point after BrdU washout, and subjected to staining and FACS analyses. (G) BrdU-positive cells were identified, and DNA contents of these cells were quantified by 7-AAD intensity. A peak with a low 7-AAD intensity represents the G1 population, and another peak with a high intensity represents G2-M populations.

(H) Live imaging analyses of FUCCI-expressing tet-shABR cells. Bottom pictures are snapshots from time-lapse tracing of single cells. t=0:00 corresponds to NEB. Scale bar represents 10 µm.

(I) Domain structure of ABR and its mutants.

The western blotting and BrdU assays were done two times (B and G). The immunostaining was repeated three times with three replicates in each experiment (D). The representative results were shown. Q-PCR experiments were repeated three times and data are shown as bar graphs (C and E). Error bars in graphs represent SD (C and E). Live imaging was performed as three independent experiments (H). Statistics: student's *t* test (C and E, $n = 3$); not significant (n. s.) and * $p < 0.05$.

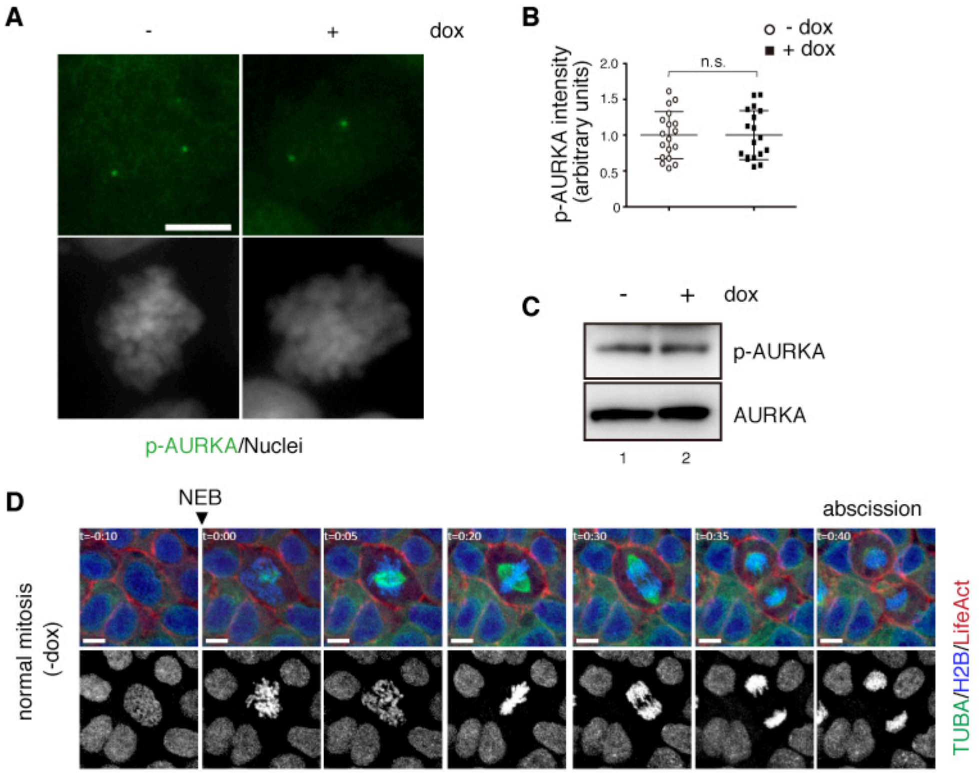

Figure S2. Abnormalities of ABR-depleted cells in centrosome separation and cell division, related to Figures 2 and 3.

(A-C) Evaluation of centrosome maturation. Cells were treated with or without dox for 3 days and phospholyration level of AURKA was examined by Immunostaining (A). Scale bar represents 10 μm. The signal intensity of paired centrosomal fluorescence was quantified by image analyses and shown as a scatter plot (B). Western blotting analyses were also done (C).

(D) A control experiment for live imaging of tet-shABR cells expressing fluorescent protein-tagged H2B (chromosome, bleu or gray), α-tubulin (TUBA, mitotic spindle, green), LifeAct (F-actin, red). t = 0 corresponds to NEB onset. Scale bars represent 10 μm.

The immunostaining was repeated three times with three replicates in each experiment (A and B). The western blotting was done three times (C). Error bars in graph represent SD (B). The imaging experiments were done three independent times. The representative examples were shown (A, C and D). Statistics: student's *t* test (B, n = 18 for control, n=18 for dox); not significant (n. s.).

A

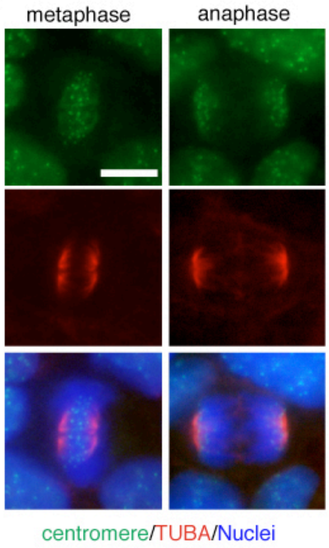

B

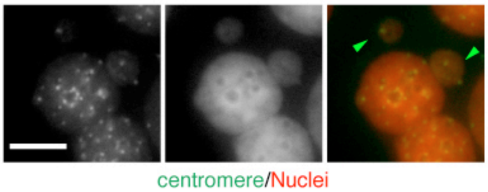

C

Chromosome number		< 46	46	47-91	92	> 92
control	sample 1	1	47	2	0	0
	sample 2	0	48	0	2	0
	sample 3	0	49	0	1	0
+dox	sample 1	3	40	4	3	0
	sample 2	7	39	2	3	0
	sample 3	5	37	5	2	1

D

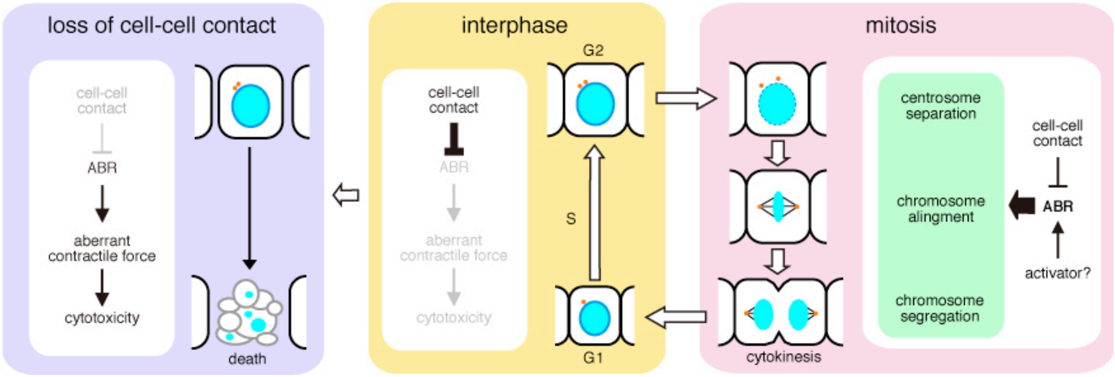

Figure S3. Chromosome segregation in ABR-depleted cells, related to Figure 4.

(A) Examples for a typical staining pattern of metaphase and anaphase cells. Centromere (green), TUBA (red), nuclei (blue). Scale bar represents 10 µm.

(B) Micronuclei in ABR-depleted cells (green arrowheads). Scale bar represents 10 µm.

(C) Summary of chromosome counting. Each mitotic spread was prepared from the cells that were treated with or without dox for 5 days, and subjected to DAPI staining. According to chromosome number, cells are categorized into the indicated five groups.

(D) Schematic diagram of ABR actions (see main text).

The immunostaining was repeated three times with five replicates in each experiment (A and B). The mitotic spreads for chromosome counting were prepared in three separated experiments (C).

Movie S1. Centrosome separation in ABR-depleted cells, related to Figure 2.

The tet-sABR hESCs expressing an mVenus-CENT2 were imaged for 12 hr. (part.1) control experiment. (part. 2) Time-lapse recording was performed in the presence of dox. t = 0 corresponds to the time point of separation initiation.

Movie S2. Mitotic progression in ABR-depleted cells, related to Figure 3.

The tet-sABR hESCs expressing a fluorescent protein-fused H2B (blue), TUBA (green), LifeActi (red) were imaged for 48 hr. (part. 1) control experiment. (part. 2~4) Time-lapse recording was performed in the presence of dox. Examples for cell death (part 2), cytokinesis failure (part 3) and extended mitosis (part. 4) are shown. t = 0 corresponds to NEB onset.

Movie S3. Chromosome segregation errors in ABR-depleted cells, related to Figure 4.

The tet-shABR hESCs expressing ECFP-fused H2B were imaged in the presence of dox. t = 0 corresponds to NEB onset.

SUPPLEMENTAL EXPERIMENTAL PROCEDURES

Cell culture.

All experiments using hESC lines were approved by an institutional ethics committee and done following the hES cell guidelines of the Japanese government. Undifferentiated hESCs were cultured on feeder layers of mouse embryonic fibroblasts (MEF; purchased from Kitayama Labes) in D-MEM/F12 (Sigma) supplemented with 20% KSR additive, 2 mM glutamine, 0.1 mM non-essential amino acids (Invitrogen), 5 ng/ml recombinant human bFGF (Wako) and 0.1 μM 2-ME under 2% CO_2. For cell passaging, hESC colonies were detached and recovered *en bloc* from the feeder layer by treating them with CTK dissociation solution at 37°C for 5-7 minutes, followed by tapping the cultures and flushing them with a pipette. The detached ESC clumps were broken into smaller pieces by gently pipetting them several times and then these small clumps were transferred onto a MEF-seeded dish. For feeder-free cultures, contaminating MEF cells were removed by incubating the cell suspension on a gelatin-coated plate at 37°C for 2 hours in the maintenance culture medium. The MEF-free hESCs were seeded and maintained on Matrigel substrate (BD Biosciences) in MEF-conditioned medium. The culture medium was refreshed daily until the next passage.

Plasmids and transfection

The cDNAs for *TUBA* and *CENT2* were amplified by PCR using PrimeSTAR GXL (Takara) using KhES-1 cDNA as a template. The generation of ABR mutants was described previously (Ohgushi et al, 2010). The LifeAct-TagRFP vector was purchased from Idibi. A lentivirus vector for FUCCI2 reporter was a gift from Dr. Miyawaki (RIKEN, BRC). The cDNAs for other fluorescence protein-fused proteins were obtained by PCR. All cDNAs were subcloned into the *pENTR/D* entry vector (Invitrogen) and subsequently sequenced. To generate stable cell lines, cDNAs were subcloned to piggybac transposon vectors containing a CAG-promoter driving expression cassette followed by an IRES-NeoR or an IRES-PuroR cassette.

The transfection to hESC with cDNA expression plasmids was performed with the FuGENE HD transfection reagent (Roche), as described previously (Ohgushi et al., 2010). To obtain stable transfectants, the cDNA expression cassettes were integrated into genomes using a piggybac transposon (PB) system. The PB vectors were co-transfected into hESC with a *pCAG-PBase* expression vector (Ohgushi et al., 2015). A few days after the transfection, cells were passaged to DR4 MEF (Cell Systems)-coated dishes and, on the following day, the medium was switched to a 100 μg/ml G418 or a 2 mg/ml puromycin-containing one. To avoid clone biases, we used the stable transfectants as a drug-resistant pool. In the case of introducing multiple transgenes, *PB-CAG-INeo* and *PB-CAG-IPuro* vectors were co-transfected and stable pools with both G418- and puromycin-resistance were selected.

For inducible ABR knockdown, we used the Tet-inducible shRNA expression lentivirus vector system. The preparation of lentivirus vectors, production of recombinant lentiviruses, infection to hESCs and FACS sorting were performed as previously described (Ohgushi et al., 2015). To induce shRNA expression, the culture medium was switched to a fresh one containing 1 μg/ml of dox, and the medium was changed daily until the analyses were completed. Note that, in the analyses of dox-treated samples,

dead or detached cells that emerged during the culture were excluded from the assay, because they were washed-out during the medium change.

Immunostaining, western blot analyses and quantitative real-time PCR

Immunostaining was performed as previously described (Watanabe et al., 2007). The cells were seeded onto a MEF-coated 8-well chamber slide, and fixed with 4% PFA at 4°C for 20 minutes and then permeabilized with 2% Triton-X100 solution. After incubation with blocking solution (2% skim milk), cells were incubated in the blocking solution containing specific antibodies. The staining was visualized using secondary antibodies conjugated with AlexaFluor-488, -546 or -647 (Invitrogen). Experiments were performed at least three times. Antibodies used in this work are listed below. For F-ACTIN staining, AlexaFluor-conjugated phalloidin (Invitrogen) was used. Nuclei were stained with DAPI or DRAQ5 (Cell Signaling). For analyses of metaphase-arrested cells, cells were treated with 1 μg/ml MG132 for 1 hr and then immediately subjected to immunostaining. Images were obtained with a fluorescence microscopy (AxioCam, Zeiss) or an inverted confocal microscopy (LSM780, Zeiss).

For the detection of endogenous protein expression in hESCs, cells were transferred onto Matrigel to minimize the possible contamination of MEF-expressing proteins into hESC lysates. After dox treatment, cells were washed with PBS, treated on the plate with HEPES lysis buffer (50 mM HEPES pH 7.4, 150 mM NaCl, 1 mM EDTA, 1 % NP-40 and protease inhibitor cocktail) for 10 min at 4°C with gentle shaking, and total cell extracts were harvested by pipetting. Immediately after adding the appropriate amount of 4 x SDS sample buffer to the extracts, they were subjected to a brief sonication for complete lysis. After boiling, the cell lysates were analyzed by SDS-PAGE and sequential western blot. A 5% skim milk solution was routinely used as a blocking reagent. Specifically, for the detection of phosphorylated proteins, 2% BSA solution was used for blocking. Images were obtained with a LAS3000 image analyzer (Fuji film).

Primary antibodies used in this work are listed below: anti-ABR (BD transduction, 611122), anti-ACTIN (Sigma, A5060), anti-p-AURKA (Cell Signaling, 3079), anti-centromere protein (Antibodies Incorporated, 15-235), HSC70 (Santa Cruz, sc-7298), anti-NANOG (R&D, AF1997), anti-OCT3/4 (BD Transduction, 611202), αTUBULIN (Millipore, MAB1864) and γTUBULIN (Sigma, T3559).

To evaluate mRNA expression, we performed quantitative PCR analyses. Total RNA was extracted using the RNAeasy Mini Kit (Quiagen) and then cDNAs were synthesized by SuperScript II reverse transcriptase (Invitrogen). The PCR reaction mixture was prepared on 96-well plate using a *Power* SYBR Green PCR Master Mix according to the manufacturer's instructions (Applied Biosystems). They were run in duplicate on a 7500 Fast Real-Time PCR System (Applied Biosystems). Expression level of each mRNA was estimated according to the corresponding standard curve and normalized to *GAPDH*. Data were displayed as arbitrary units or as relative values compared to each control. Primer sets used in this work are described in our previous paper (Ohgushi et al., 2015).

Cell cycle analyses.

To label replicating cells, cells were supplemented with BrdU and cultured for 40 min. After washing with PBS, the labeled cells were dissociated, harvested and stained using APC BrdU Flow Kit (BD

Pharmingen). The stained cells were analyzed by a Flow cytometer (BD Bioscience). To trace the cell cycle progression, the S-phase population was labeled by a transient supplementation of Brd-U. After complete washout of Brd-U, these cells were kept on culture to progress into the G2-M phase. Cells were harvested at 0, 6, 9 and 12 hours after labeling and DNA content was analyzed to trace the fate of Brd-U-positive cells. DNA contents were quantified by simultaneous staining with a 7-AAD DNA dye. Data were processed using FlowJo software (ver.12).

Karyotype analyses.

The dox-treated or -untreated cells were harvested after 2 hours treatment with 0.06 μg/ml Colcemid (Gibco). The cells were incubated in Buffered Hypotonic Solution (Genial Genetics) for 10 min, fixed by multiple changes of 3:1 methanol:acetic acid mixture, and then dropped onto dried glass slides. The condensed chromosomes are visualized by DAPI staining, and counted under the microscope. Since chromosome identification was relatively difficult in the case of dox-treated samples due to contamination of dead cell-derived DNA debris, one control and two dox-treated samples were analyzed by a professional (Chromosome Science Lab) and confirmed to obtain the identical results. To identify each chromosome, the slides were analyzed by multi-color fluorescence in situ hybridization (mFISH) using a 24XCyte Multi Color Probe Kit (MetaSystems). Probe hybridization was done using a VP2000 Processor (Abbott). Images were obtained with an MSearch imaging system and processed with ISIS software (MetaSystems).

Live imaging.

For live imaging, hESC clumps were seeded onto a MEF-coated 35-mm μ-dish (Ibidi), and they were imaged on an inverted microscope (IX81-ZDC, Olympus) that was equipped with a stepper filter wheel (Ludl) and a cooled EM-CCD camera (ImagEM, Hamamatsu Photonics). For confocal observations, serial images were collected using a CSU-W1 unit (Yokogawa) configured with an IX81-ZDC microscope. To observe centrosome behaviors, 8 images were obtained with 0.25 μm intervals along the Z-plane. Time-lapse recording was started after the 60 h dox treatment and was done for the following 12 h with 2-min time intervals. In the case of the observation of mitotic progression, 10 images were obtained with 1-μm intervals along the Z-plane. After 24 h dox treatment, recording was done for the following 46 h with 5-min time intervals. In both cases, NEB is identified as a shift of non-centrosomal fluorescence signal from cytosolic to diffuse pattern. The maximum projection image was constructed from the obtained slices using MetaMorph software.

Statistical analyses.

Error bars in the figures represent standard deviations. Statistical significance was tested by Student's t-test for two-group comparison, and by one-way ANOVA for multi-group comparison with Dunnett's test using Prism4 software (GraphPad).

SUPPLEMENTAL REFERENCES

Ohgushi, M., Minaguchi, M. and Sasai, Y. (2015). Rho-signaling-directed YAP/TAZ activity underlies the log-term survival and expansion of human embryonic stem cells. Cell Stem Cell *17*, 448-461.

Ohgushi, M., Minaguchi, M. and Sasai, Y. (2015). Rho-signaling-directed YAP/TAZ activity underlies the log-term survival and expansion of human embryonic stem cells. Cell Stem Cell *17*, 448-461.

Watanabe, K., Ueno, M., Kamiya, D., Nishiyama, A., Matsumura, M., Wataya, T., Takahashi, J.B., Nishikawa, S., Nishikawa, S., Muguruma K., et al. (2007). A ROCK inhibitor permits survival of dissociated human embryonic stem cells. Nat. Biotechnol. *25*, 681-686.
