# Supplementary figures and images for: A RHO Small GTPase Regulator ABR Secures Mitotic Fidelity in Human Embryonic Stem Cells
Source: Stem Cell Reports. 2017 Jun 1;9(1):58–66. doi: 10.1016/j.stemcr.2017.05.003 (PMC5511046; doi:10.1016/j.stemcr.2017.05.003)

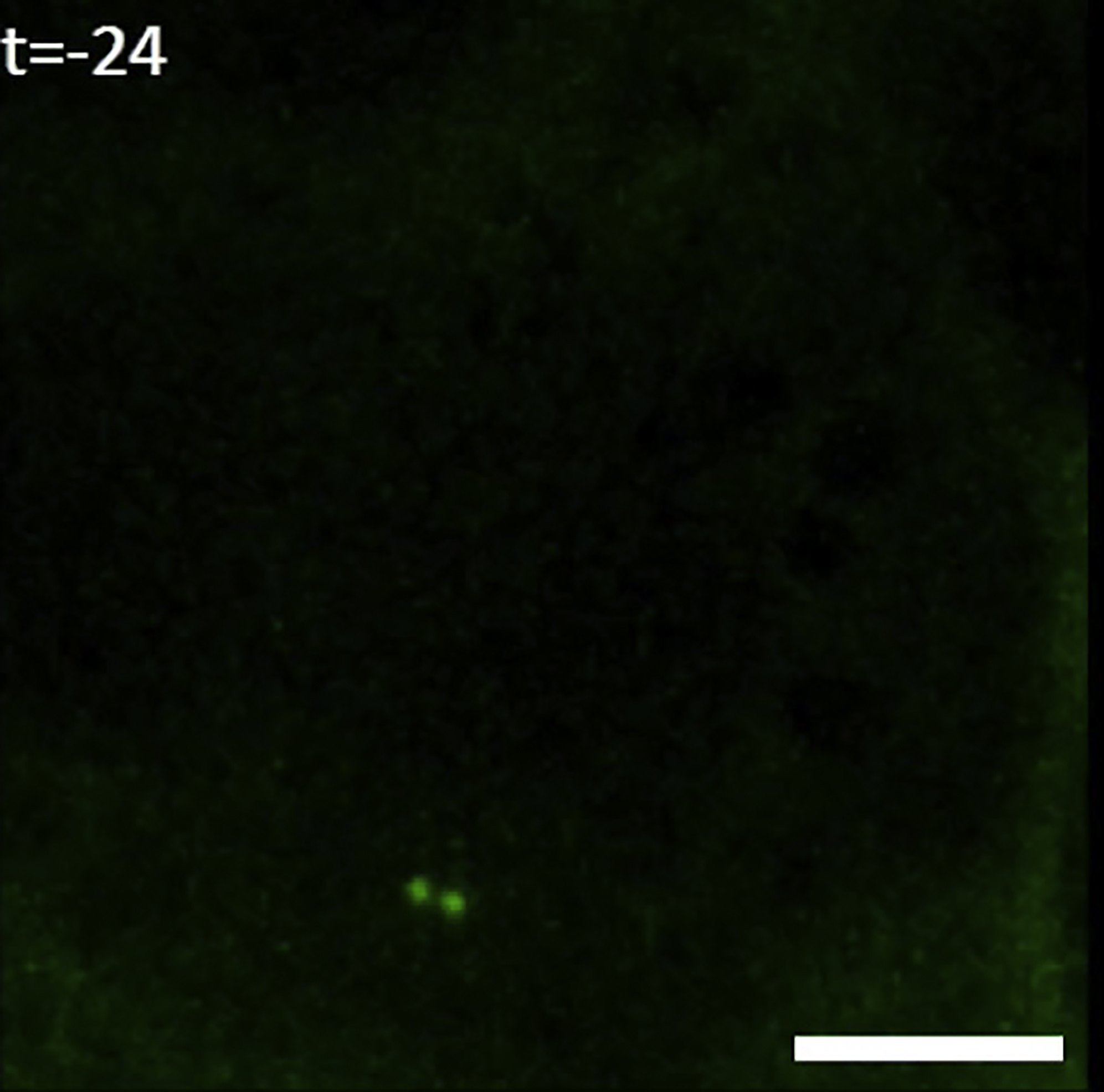

Supplement: Movie S1. Centrosome Separation in ABR-Depleted Cells, Related to Figure 2 [file mmc2.jpg]

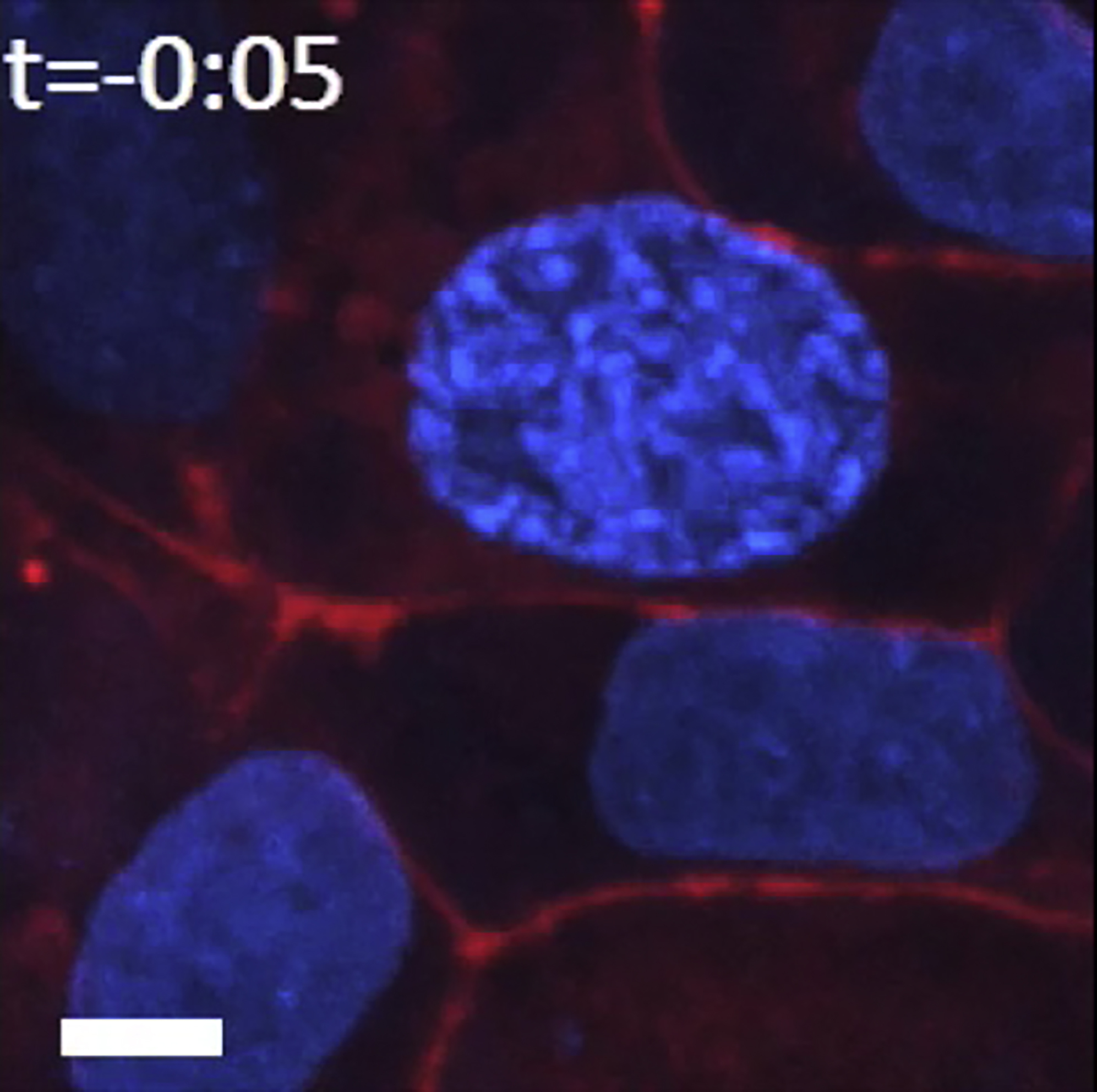

Supplement: Movie S2. Mitotic Progression in ABR-Depleted Cells, Related to Figure 3 [file mmc3.jpg]

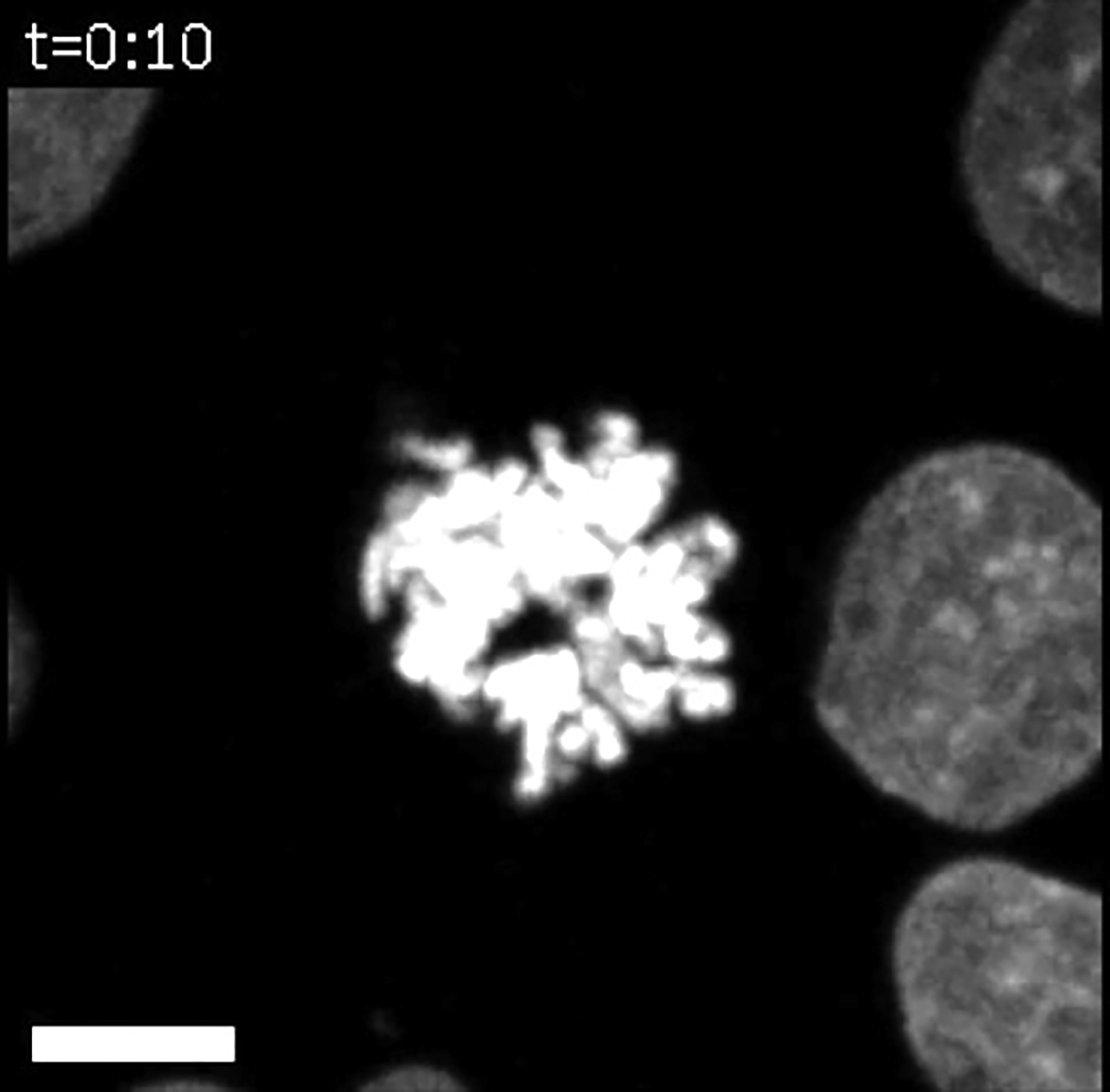

Supplement: Movie S3. Chromosome Segregation Errors in ABR-Depleted Cells, Related to Figure 4 [file mmc4.jpg]
