# Supplementary material for: A RHO Small GTPase Regulator ABR Secures Mitotic Fidelity in Human Embryonic Stem Cells
Source: Stem Cell Reports. 2017 Jun 1;9(1):58–66. doi: 10.1016/j.stemcr.2017.05.003 (PMC5511046; doi:10.1016/j.stemcr.2017.05.003)
Supplement: Document S2. Article plus Supplemental Information [file mmc5.pdf]

Stem Cell Reports
Report
OPEN ACCESS

A RHO Small GTPase Regulator ABR Secures Mitotic Fidelity in Human Embryonic Stem Cells

Masatoshi Ohgushi,[1,2,3,*] Maki Minaguchi,[1,2] Mototsugu Eiraku,[3,4] and Yoshiki Sasai[1,2,5]
[1]Human Stem Cell Technology Unit
[2]Laboratory for Organogenesis and Neurogenesis
[3]Laboratory for in Vitro Histogenesis
RIKEN Center for Developmental Biology, Kobe 650-0047, Japan
[4]Laboratory for Developmental Systems, Institute for Frontier Life and Medical Science, Kyoto University, Kyoto 606-8507, Japan
[5]Deceased August 5, 2014
*Correspondence: mohgushi@cdb.riken.jp
http://dx.doi.org/10.1016/j.stemcr.2017.05.003

SUMMARY

Pluripotent stem cells can undergo repeated self-renewal while retaining genetic integrity, but they occasionally acquire aneuploidy during long-term culture, which is a practical obstacle for medical applications of human pluripotent stem cells. In this study, we explored the biological roles of ABR, a regulator of RHO family small GTPases, and found that it has pivotal roles during mitotic processes in human embryonic stem cells (hESCs). Although ABR has been shown to be involved in dissociation-induced hESC apoptosis, it does not appear to have direct effects on cell survival unless cell-cell contact is impaired. Instead, we found that it is important for faithful hESC division. Mechanistically, ABR depletion compromised centrosome dynamics and predisposed the cell to chromosome misalignment and missegregation, which raised the frequency of aneuploidy. These results provide insights into the mechanisms that support the genetic integrity of self-renewing hESCs.

INTRODUCTION

The faithful inheritance of genetic material during repetitive cell division is fundamental for animal development and tissue regeneration in multicellular organisms. Several quality control mechanisms survey the organism for genetic normality and then activate programs for error correction or elimination of abnormal cells. These mechanisms could suppress aneuploidy, a genetic aberration that arises from missegregation of whole chromosomes during mitosis. If aneuploid cells override these barriers and continue proliferating, they can acquire cancerous properties. It is well recognized that chromosomal instability, the condition in which aneuploidy occurs at a high rate, underlies genetic abnormalities found in many types of tumor cells. Actually, aneuploidy is commonly observed in a wide range of tumor tissues and cancer-derived cell lines (reviewed in Santaguida and Amon, 2015).

Pluripotent stem cells, such as embryonic stem cells (ESCs) and induced pluripotent stem cells (iPSCs), have special abilities to differentiate into cells of all three germ layers (pluripotency) and to undergo unlimited proliferation while retaining their identities (self-renewal) (Nichols and Smith, 2012). In addition, they are known to be able to maintain genetic integrity, which is an essential requirement for their utilization in genetic studies or medical applications. Maintaining chromosome number is particularly important in pluripotent stem cells because aneuploidy can lead not only to oncogenic transformation but also to differentiation dysregulation (Peterson and Loring, 2014; Ben-David et al., 2014; Lamm et al., 2016; Zhang et al., 2016). Nevertheless, aneuploidy is often observed in some human ESC (hESC) and iPSC lines (Spits et al., 2008; Mayshar et al., 2010; Taapken et al., 2011). A screening study of a large number of hESC/iPSC lines documented a progressive tendency to acquire karyotypic abnormality during long-term culture, indicating a culture-associated susceptibility to aneuploidy (International Stem Cell Initiative et al., 2011). Although previous reports describe several putative risks contributing to chromosome instability, including excessive replication stresses and DNA damage responses (Zhao et al., 2015; Lamm et al., 2016; Jacobs et al., 2016), safeguarding mechanisms to counteract these threats remain to be elucidated.

We previously reported that the aberrant activation of the RHO-ROCK pathway was responsible for dissociation-induced hESC apoptosis (Watanabe et al., 2007; Ohgushi et al., 2010). We also identified ABR, a modulator of RHO family small GTPase activities, as an upstream factor controlling the survival-or-death decision of dissociated hESCs. The ROCK activation is thought to affect cellular motility (Li et al., 2010), but whether this phenomenon represents any biological implications has remained a mystery. To tackle this question, we sought to explore ABR function. We found that ABR did not have direct effects on cell survival unless cell-cell contact was impaired. Instead, we obtained unexpected data indicating that ABR depletion increased the frequency of chromosome missegregation. These findings shed light on the safeguarding

 © 2017 The Authors.
This is an open access article under the CC BY license (http://creativecommons.org/licenses/by/4.0/).

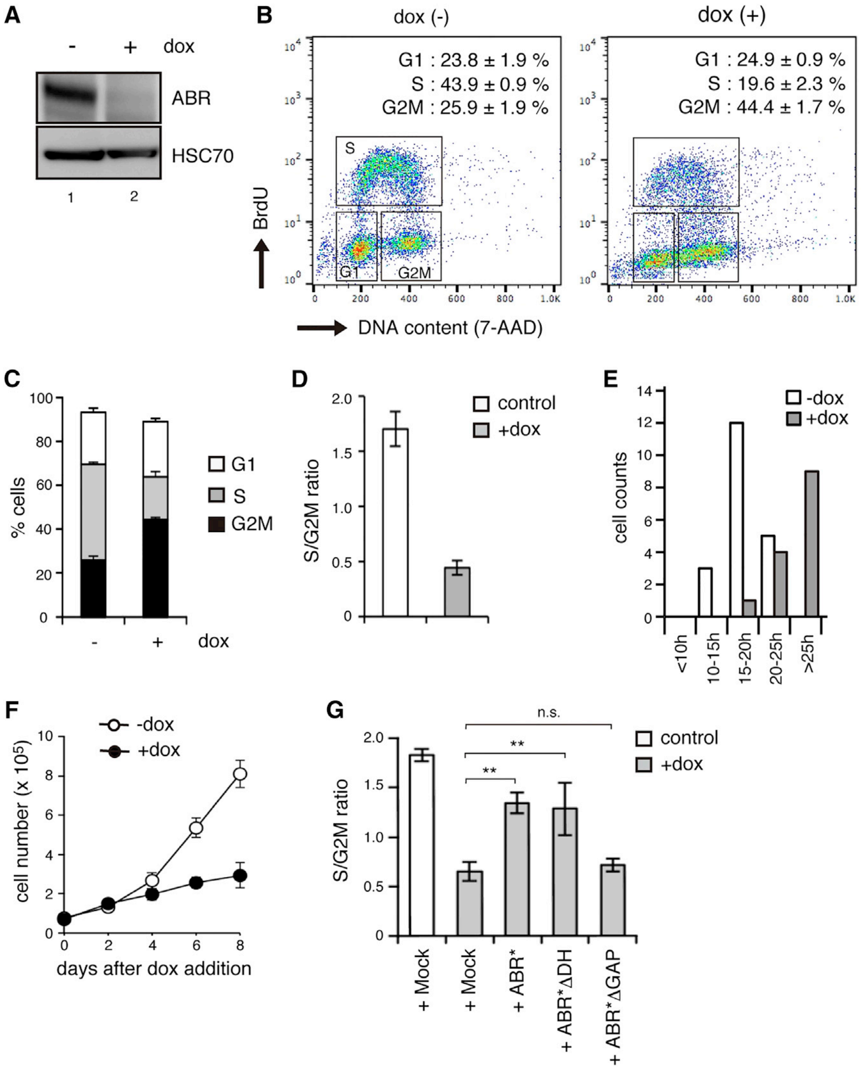

Figure 1. ABR Depletion Leads to G2-M Accumulation

(A) Western blotting analyses. The tet-shABR cells were cultured with or without dox for 3 days. HSC70 was examined as a loading control.

(B and C) Cell cycle profile of dox-treated (right) or untreated (left) tet-shABR cells. The histograms show representative results from three independent experiments (B). The occupancy of each phase in the analyzed cells is indicated in these histograms and also shown as a bar graph (C).

(D) Population dominance of S versus G2-M phase is represented as the ratio of S to G2-M phase cells.

(E) FUCCI-expressing tet-shABR cells were cultured with or without dox for 72 hr. Cells were classified into the indicated five categories according to the time length of one round of cell cycle (n = 20 from three independent imaging experiments).

(F) Growth curve of tet-shABR cells that were cultured with or without dox for 8 days.

(G) Rescue experiments. The expression of RNAi-resistant ABR mutants (Abr*) lacking the GEF domain (ΔDH) but not GAP domain (ΔDH) in ABR-depleted hESC restores S phase dominance. ABR* was used as a positive control.

All experiments were repeated three times and data are shown as representative (A and B), bar graphs (C, D, and G), or a scatterplot (F). Error bars in graphs represent SD (C, D, F, and G). Statistics: Dunnett's test (G, n = 3) versus lane 2; n.s., not significant and $^{**}p < 0.05$. See also Figure S1.

mechanism that prevents chromosomal instability in hESCs.

RESULTS

ABR Depletion Caused Cellular Accumulation at the G2-M Phase of the Cell Cycle

To examine ABR functions in hESCs, we applied a doxycycline (dox)-inducible short hairpin RNA expression strategy (Figure S1A, and refer to Ohgushi et al., 2015). This method permitted the selective depletion of target molecules with controlled timing and under the same genotypic background. We succeeded in reducing ABR protein to an undetectable level after dox addition (Figure 1A), and we refer to these genetically engineered cells as tet-shABR hESCs. To address the putative primary responses caused by ABR depletion, we first examined cellular behaviors on day 3 of dox treatment when the ABR protein level seemed to reach a minimum (Figure S1B). The expression levels of pluripotent markers were nearly equal between control and dox-treated cells (Figures S1C and S1D). The number of dead cells significantly increased after dox treatment, but the extent was not substantial (Figure S1E). At this time point, it was the cell cycle profile that we found remarkably different between dox-treated and untreated tet-shABR cells (Figures 1B and 1C).

ESCs are known to exhibit a characteristic cell cycle pattern that includes an abbreviated G1 phase and dominant occupancy of replicating S phase cells (Boward et al., 2016). Indeed, our control cells exhibited this typical pattern (Figure 1B, left). Interestingly, in dox-treated cells, the S phase population was decreased while the G2 and M populations were greatly increased (Figure 1B, right),

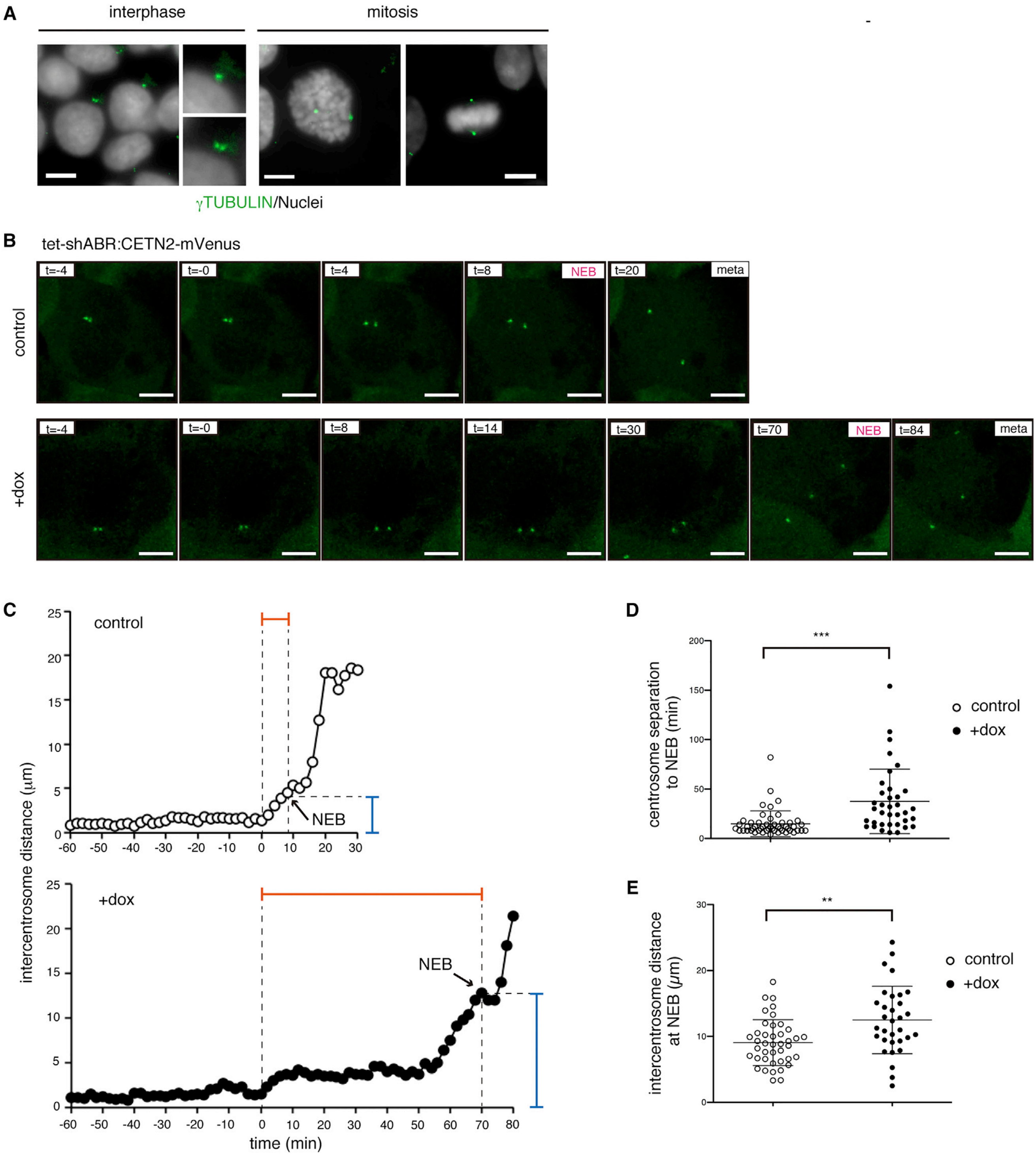

Figure 2. ABR Controls Centrosome Dynamics

(A) Centrosomes are visualized by γTUBULIN staining (green). Mitosis or interphase is determined by chromosomal staining pattern and morphology (gray).

(B–E) Live imaging analyses of control or dox-treated tet-shABR cells expressing mVenus-CENT2. (B) Snapshots from Movie S1. The distance between centrosomes was measured with 2-min intervals. t = 0 corresponds to separation starting time, defined as a no-return point of bilateral movement. Arrows indicate NEB onsets. (D) The durations from separation initiation to NEB. The y axis corresponds to the

(legend continued on next page)

resulting in an inversion in population dominancy (Figures 1C and 1D). These observations demonstrate that ABR-depleted hESCs accumulate at the G2-to-M stage. To further confirm this, S phase cells were labeled with a transient bromodeoxyuridine supplementation, and then traced during the subsequent 12 hr (Figures S1F–S1G). In control cells, the labeled population passed through G2-M into the next G1 phase. In dox-treated cells, however, labeled cells seemed to be trapped at the 4N state and struggled to proceed into the next cycle, suggesting that ABR-depleted cells had trouble entering or exiting mitosis. In addition to these population analyses, we performed single-cell tracing using tet-shABR cells expressing a FUCCI reporter (Figure S1H, Sakaue-Sawano et al., 2008). This revealed the tendency of ABR-depleted cells to take longer times to complete one round of a cell cycle than did control cells (Figure 1E). Consequently, ABR-depleted cells showed significant growth retention when cultured for a further extended period (Figure 1F).

ABR protein has a unique domain structure: a guanine nucleotide exchanging factor (GEF) domain at the N terminus and a GTPase-activating protein (GAP) domain at the C terminus (Figure S1I). When isolated and tested by in vitro assay, these domains were shown to possess GEF and GAP activities for the selected members of RHO family small GTPases (Heisterkamp et al., 1993; Chuang et al., 1995). We sought to determine which domain is responsible for ABR's ability to drive cell cycle progression by restoring ABR expression using RNAi-resistant or domain-deleted mutants (Figure 1G). The introduction of codon-swapped RNAi-immune mutant (ABR*) into tet-shABR hESCs restored the S phase dominance. A partial restoration was observed when an ABR mutant lacking a GEF domain was introduced. On the other hand, a GAP-dead mutant showed little rescuing effects, indicating the importance of GAP activity for ABR.

In sum, these results show that ABR plays a key role in cell cycle progression from G2-M to the next G1 phase through its GAP activity.

Compromised Centrosome Dynamics upon ABR Depletion

To obtain mechanistic insights into the accumulation of ABR-depleted cells in the G2-M phase, we focused on the centrosome, a central organelle that operates multiple mitotic events (Tanenbaum and Medema, 2010). Centrosomes were replicated during S phase, matured at G2 phase and separated bilaterally in parallel with M phase entry (Figure 2A), all of which are important prerequisites for proper cell division. In both control and dox-treated cells, duplicated centrosomes were evident at prophase (data not shown). The phosphorylation level of a centrosomal kinase AURORA-A (AURKA) did not demonstrate a substantial difference in centrosome maturation (Figures S2A–S2C). Otherwise, by monitoring centrosome dynamics using tet-shABR hESCs expressing mVenus-fused centrin-2 (CETN2), a component of the centrosome, we found that it took longer in dox-treated cells for each centrosome to move to the opposite side (Figure 2B and Movie S1). Notably, whereas in control cells the nuclear envelope breakdown (NEB) occurred immediately after centrosomes started to move bilaterally, a much longer time was needed for NEB to take place in dox-treated cells (Figures 2B and 2C). As a consequence, inter-centrosomal distances at the time of NEB were significantly increased in ABR-depleted cells (Figure 2D). These observations raise the possibility that anomalous centrosome behaviors could be a mechanistic link between ABR dysfunction and G2-M accumulation. In support of this idea, it has been reported that RAC, a downstream small GTPase of ABR, modulates centrosome movement during G2-to-M progression in cultured epithelial cells (Woodcock et al., 2010; Whalley et al., 2015).

Multiple Mitotic Failures in ABR-Depleted hESCs

A number of previous studies indicate that compromised centrosome separation often leads to severe failures in mitotic processes (Nam et al., 2015). To observe mitosis processes in cells with reduced ABR expression, we monitored cell cycle progression using tet-shABR hESCs expressing fluorescence protein-fused H2B (a marker for chromosomes), α-tubulin (TUBA, a marker for mitotic spindle) and Lifeact (a marker for actin filament) (Figures 3A–3C, Movie S2, part 1 and Figure S2D for a control experiment). Through these live imaging studies, we first found that a substantial number of dox-treated cells faced unrecoverable mitotic errors, including cell death or cytokinesis failures (Figures 3A, 3B and 3D; Movie S2, parts 2 and 3). Most of these cells had encountered problems in chromosomal alignment before these serious errors. Looking into these data more carefully, we also found that the majority of dox-treated cells struggled to align chromosomes at the central plane and spent significantly extended times before exiting from mitosis, even if they were finally able to divide (Figures 3C and 3E; Movie S2, part 4). In addition to these

red line-gated periods indicated in (C) control (n = 50) and dox-treated cells (n = 36) were analyzed. (E) The distance between centrosomes at the time of NEB. The y axis corresponds to blue line-gated lengths indicated in (C) control (n = 41) and dox-treated cells (n = 32) were analyzed.
The imaging experiments were performed three times. Scale bars represent 10 μm. Error bars in the graphs represent SD (D and E). Statistics: Student's t test (D and E); ***$p < 0.001$ and **$p < 0.01$. See also Figure S2 and Movie S1.

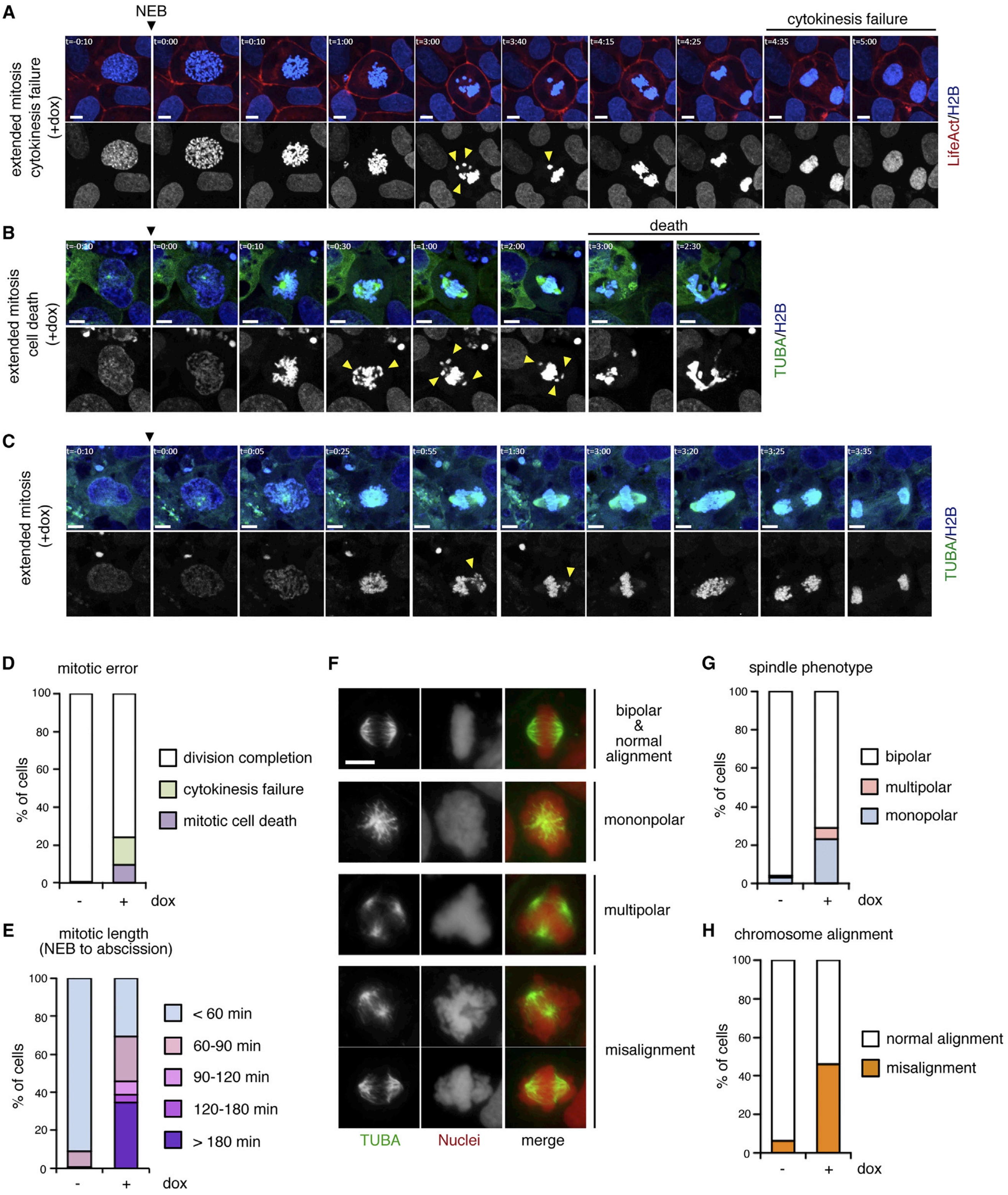

Figure 3. Multiple Mitotic Failures upon ABR Depletion

(A–E) Snap shots from Movie S2. Live imagings were performed using dox-treated tet-shABR cells expressing fluorescent protein-tagged H2B (chromosome, blue or gray), α-tubulin (TUBA, mitotic spindle, green) and LifeAct (F-actin, red). Examples of cytokinesis error (A),

(legend continued on next page)

live imaging data, our immunostaining analyses using metaphase-arrested cells showed a high frequency of spindle malformation, which might arise from defects in centrosome separation, as well as misaligned chromosomes in dox-treated cells (Figures 3G and 3H).

Thus, ABR-depleted cells encountered serious difficulties in chromosome alignment at metaphase, which delayed their transition into anaphase. This might be another cause for the accumulation of ABR-depleted cells in the G2-M stage (Figure 1B).

Chromosomal Missegregation and Aneuploidy in ABR-Depleted hESCs

Despite these troubles during prophase or metaphase, a large fraction of ABR-depleted cells did proceed to anaphase. This indicates that ABR is not absolutely required for hESCs to complete cell division. The extended period for metaphase-to-anaphase transition implies the activation of salvage mechanisms that serve as a backup when normal processes are disrupted (Musacchio, 2015). In these cases, however, we repeatedly observed lagging chromosomes during anaphase-to-telophase progression and a resultant micronucleus formation in the daughter cells (Figure 4A; Movie S3). Immunostaining analyses revealed that ABR depletion increased the incidence of these signs for chromosome missegregation (Figures 4B, 4C, and S3B; S3A shows typical staining patterns). From these data, we speculated that hESCs are able to bypass a mitotic necessity of ABR with the help of salvage mechanisms, but this process renders the cell susceptible to erroneous chromosome segregation.

We postulated that such an error-prone situation would yield a selective pressure to facilitate the emergence of aneuploid cells. With this in mind, we carefully examined chromosome counts in the mitotic spreads that were prepared from cells treated with dox for 5 days, because at this time point most cells might undergo a few rounds of division in an ABR-independent way. Consistent with a previous report showing that the hESC line used here is stable in the karyotype during long-term culture (International Stem Cell Initiative et al., 2011), most of the control cells retained the normal number of chromosomes (Figure 4D). On the other hand, when cultured with reduced ABR expression, hESCs showed abnormal karyotypes with a higher frequency (Figures 4D and S3C). Some of them were tetraploid, which can result from mitosis skip or cytokinesis failure, and notably, others showed a gain or loss of some chromosomes (Figures 4E and S3C). Thus, ABR dysfunction actually elevated the risk of aneuploidy, highlighting a pivotal role of ABR in preventing aneuploidy in cultured hESCs.

DISCUSSION

In this study, we explored ABR function in clump-cultured hESCs. We first noticed that ABR depletion impeded G2-to-M-to-G1 transitions. Deeper investigations at a single-cell level revealed that ABR-depleted cells struggled to complete a couple of mitotic steps, including centrosome separation at prophase and chromosome alignment at metaphase. These observations indicated that ABR has a crucial role in mitosis progression. Important information lacking now is subcellular localization of ABR. Our attempts to determine its localization in hESCs did not work well, but a large-scale proteomics analysis demonstrated ABR as a putative interactor of some centrosomal proteins (e.g., CEP25, Fogeron et al., 2013), supporting our conclusion.

ABR seems to play a safeguarding role in mitotic fidelity, in addition to being an apoptosis promoter in dissociated cells (Figure S3D), and these different outcomes upon ABR activation are dictated by the cellular adhesive state, dissociation versus clumping. A previous report demonstrated that the mitotic activation of actomyosin sometimes stimulated cell death, mirroring the dissociation-induced phenotype (Barbaric et al., 2014). Considering that cellular adhesiveness is dynamically rearranged during mitosis, spontaneous failures in the adhesion-mediated control of ABR activity could occur upon mitosis. An intriguing possibility is that mitotic cells in which ABR is inappropriately regulated might be intrinsically programmed to be eliminated, representing a mechanism

cell death (B), and extended mitosis (C) are shown. Yellow arrowheads indicate misaligned chromosomes. (D) The incidence of mitotic errors. Cellular behaviors were categorized into the indicated three groups. Control (n = 146) and dox-treated cells (n = 87) were analyzed.
(E) The mitosis duration. In the cells that progressed into the next stage (classified as “division completion” in D), the time length from NEB to abscission was categorized into the indicated five groups. Control (n = 144) and dox-treated cells (n = 72) were analyzed.
(F) Immunostaining analyses on metaphase-arrested tet-shABR cells that were treated or untreated with dox for 3 days. Nuclei (gray or red) and TUBA (green) are shown. According to spindle morphology or chromosome positions, cellular phenotypes were classified into the indicated five categories.
(G and H) The incidence of spindle malformation (G) and chromosome misalignment (H). Control (n = 94) and dox-treated cells (n = 52) were analyzed.
The imaging experiments were repeated three times, and representative examples were shown (A, B, and C). The immunostaining was performed two times with three replicates in each experiment (F). Scale bars represent 10 μm. See also Figure S2 and Movie S2.

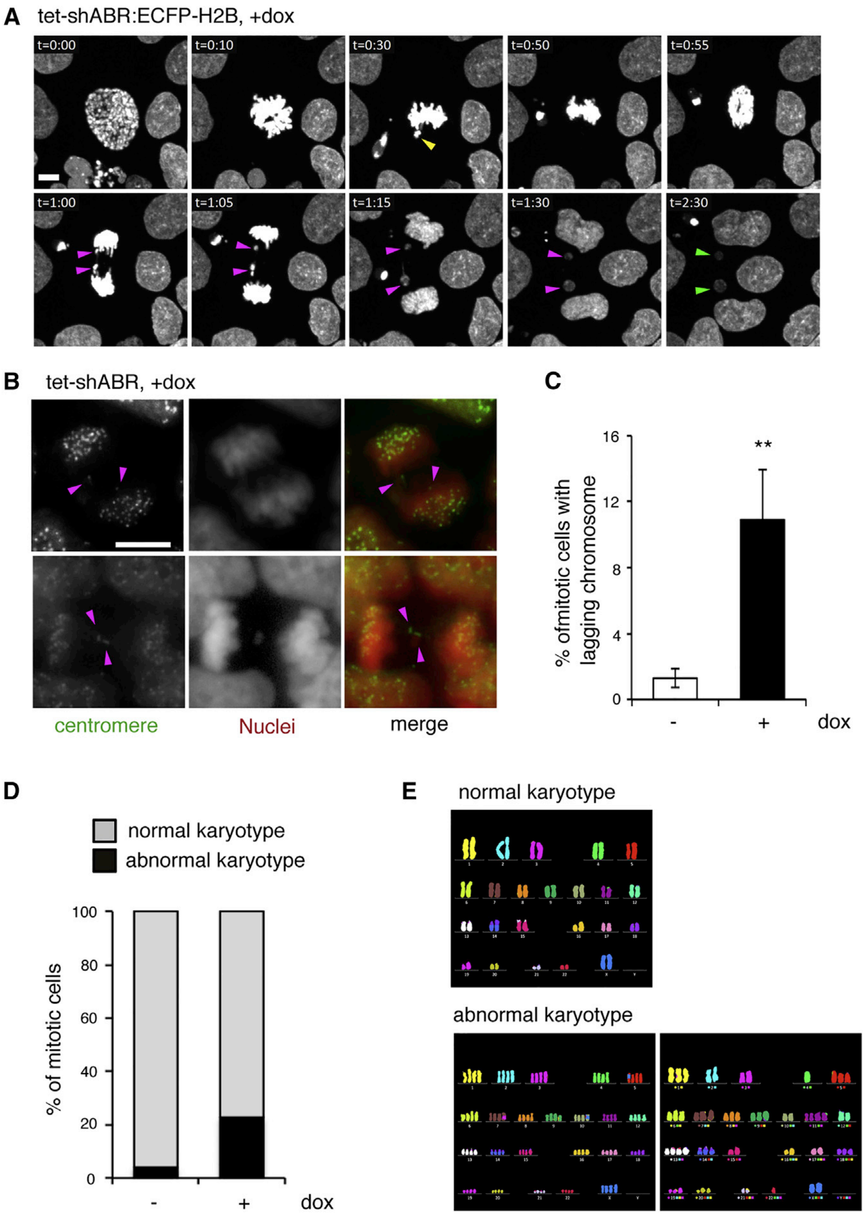

Figure 4. Chromosome Missegregation and Aneuploidy in ABR-Depleted Cells

(A) Snapshots from Movie S3. Yellow, magenta, and green arrowheads indicate misaligned chromosomes at metaphase, lagging chromosomes at anaphase, and micronuclei in daughter cells, respectively.
(B and C) Immunostaining analyses for lagging chromosomes. Two representatives from dox-treated samples are shown in (B) (nuclei, red; centromeres, green). Magenta arrowheads indicate centromere-positive lagging chromosomes. Mitotic cells with lagging chromosomes were counted and the incidence was shown in (C). Control (n = 315) and dox-treated cells (n = 318) were analyzed.
(D) Chromosome counting analyses. Mitotic spreads were prepared using tet-shABR cells that were treated or untreated with dox for 5 days. In each sample, 50 mitotic cells were subjected to counting.
(E) Multicolor fluorescence in situ hybridization (FISH) analyses. The dox-treated sample was stained with FISH probes for each chromosome. Two independent experiments were performed, and representative examples for normal and abnormal karyotypes are shown.
The immunostaining was repeated three times with five replicates in each experiment (B and C). The mitotic spreads for chromosome counting were prepared in three separate experiments (D). Scale bars, 10 μm. Error bars in the graphs represent SD. Statistics: Student's t test (C, n = 3); $^{**}p < 0.05$. See also Figure S3 and Movie S3.

restraining expansion of genetically abnormal cells. Consistently, it seems that ABR is not absolutely required for mitosis completion, but mitosis without ABR is an error-prone process leading to frequent chromosome missegregation. These results indicate that ABR sets a robust way for chromosome segregation in hESCs. This might be favorable, particularly to the self-renewing pluripotent stem cells in which the postmitotic checkpoint signaling is likely uncoupled to apoptosis-mediated elimination of genetically abnormal cells (Mantel et al., 2007).

How ABR participates in the control of mitotic fidelity remains an open question. Taking into consideration that ABR action is correlated with a cellular adhesive state, the present study suggests an unrecognized link between cell-cell contact and mitotic fidelity. In general, most types of non-transformed epithelial cells stop proliferation after forming a polarized layer in confluent culture, a phenomenon known as "contact inhibition of proliferation" (McClatchey and Yap, 2012). In this regard, hESCs are an atypical cell type: they can continue active growth within densely packed polarized colonies. We previously reported that the disconnection between cell-contact and nuclear function of transcriptional cofactors YAP/TAZ allows this type of unique proliferation (Ohgushi et al., 2015). From another viewpoint, however, this unique mode of proliferation yields complex mechanical fields for mitotic cells,

because individual cells are constantly exposed to the pushing or pulling forces from contacting adjacent cells. On the basis of the observed high incidence of chromosome missegregation in ABR-depleted cells, our hypothesis is that ABR buffers the noisy mechanical cues within a multicellular society to confer robustness in the fidelity of chromosome segregation during long-term expansion of hESCs.

Unlike somatic cells in vivo, the proliferation of which is limited to several division cycles, ESCs and iPSCs undergo numerous rounds of genome replication and cell division to fulfill the quantitative demand for their practical applications. This raises concerns about the accumulation of genetic aberrations. Among them, aneuploidy is a particular threat since some types of aneuploidy confer survival or growth advantages that outcompete normal populations (Spits et al., 2008; Avery et al., 2013; Nguyen et al., 2014). Our findings provide implications for developing hESC culture methods that are better suited for human genetic studies and cell-based therapies.

EXPERIMENTAL PROCEDURES

Cell Culture

All experiments using hESC lines were approved by an institutional ethics committee and done following the hESC guidelines of the Japanese government. Undifferentiated hESCs (KhES-1, Suemori et al., 2006) were cultured on feeder layers of mouse embryonic fibroblasts in D-MEM/F12 (Sigma) supplemented with 20% KnockOut serum replacement, 2 mM glutamine, 0.1 mM non-essential amino acids (Invitrogen), 5 ng/mL recombinant human basic fibroblast growth factor (Wako), and 0.1 μM 2-mercaptoethanol. The culture medium was refreshed daily until the next passage.

Immunostaining

Immunostaining was performed as described previously (Watanabe et al., 2007). For analyses of metaphase-arrested cells, cells were treated with 1 μg/mL MG132 for 1 hr and then immediately subjected to immunostaining.

Live Imaging

For live imaging, hESC clumps were seeded onto an MEF-coated 35-mm μ-dish (Ibidi). For confocal observations, serial images were collected using a CSU-W1 unit (Yokogawa) configured with an IX81-ZDC microscope (Olympus). The maximum projection image was constructed from the obtained slices using MetaMorph software.

Statistical Analyses

All experiments were performed at least three times, and error bars in the graphs represent SDs. Statistical significance was tested by Student's t test for two-group comparison, and by one-way ANOVA for multi-group comparison with Dunnett's test using Prime4 software (GraphPad).

SUPPLEMENTAL INFORMATION

Supplemental Information includes Supplemental Experimental Procedures, three figures and three movies and can be found with this article online at http://dx.doi.org/10.1016/j.stemcr.2017.05.003.

AUTHOR CONTRIBUTIONS

M.O. conceived the project, performed experiments, and wrote the manuscript. M.M. performed experiments. M.E. helped M.O. in imaging experiments. Y.S. supervised the project.

ACKNOWLEDGMENTS

We are grateful to all members of the Sasai and Eiraku laboratory for support, discussion, and encouragement. M.O. expresses special thanks for his mentor Y.S. with tremendous respect to his legacy in science. This work was supported by grants-in-aid from MEXT (to M.E. and Y.S.) and by JSPS KAKENHI 26830088 (to M.O.).

Received: November 6, 2016
Revised: May 2, 2017
Accepted: May 2, 2017
Published: June 1, 2017

REFERENCES

Avery, S., Hirst, A.J., Baker, D., Lim, C.Y., Alagaratnam, S., Skotheim, R.I., Lothe, R.A., Pera, M.F., Colman, A., Robson, P., et al. (2013). BCL-XL mediates the strong selective advantage of a 20q11.21 amplification commonly found in human embryonic stem cell cultures. Stem Cell Rep. *1*, 379–386.

Barbaric, I., Biga, V., Gokhale, P.J., Jones, M., Stavish, D., Glen, A., Coca, D., and Andrews, P.W. (2014). Time-lapse analysis of human embryonic stem cells reveals multiple bottlenecks restricting colony formation and their relief upon culture adaptation. Stem Cell Rep. *3*, 142–155.

Ben-David, U., Arad, G., Weissbein, U., Mandefro, B., Maimon, A., Golan-Lev, T., Narwani, K., Clark, A.T., Andrews, P.W., Benvenisty, N., et al. (2014). Aneuploidy induces profound changes in gene expression, proliferation and tumorigenicity of human pluripotent stem cells. Nat. Commun. *5*, 4825.

Boward, B., Wu, T., and Dalton, S. (2016). Control of cell fate through cell cycle and pluripotency networks. Stem Cells *34*, 1427–1436.

Chuang, T.H., Xu, X., Kaartinen, V., Heisterkamp, N., Groffen, J., and Bokoch, G.M. (1995). Abr and Bcr are multifunctional regulators of the Rho GTP-binding protein family. Proc. Natl. Acad. Sci. USA *92*, 10282–10286.

Fogeron, M.L., Müller, H., Schade, S., Dreher, F., Lehmann, V., Kühnel, A., Scholz, A.K., Kashofer, K., Zerck, A., Fauler, B., et al. (2013). LGALS3BP regulates centriole biogenesis and centrosome hypertrophy in cancer cells. Nat. Commun. *4*, 1531.

Heisterkamp, N., Kaartinen, V., van Soest, S., Bokoch, G.M., and Groffen, J. (1993). Human ABR encodes a protein with GAPrac

activity and homology to the DBL nucleotide exchange factor domain. J. Biol. Chem. *268*, 16903–16906.

International Stem Cell Initiative, Amps, K., Andrews, P.W., Anyfantis, G., Armstrong, L., Avery, S., Baharvand, H., Baker, J., Baker, D., Munoz, M.B., et al. (2011). Screening ethnically diverse human embryonic stem cells identifies a chromosome 20 minimal amplicon conferring growth advantage. Nat. Biotechnol. *29*, 1132–1144.

Jacobs, K., Zambelli, F., Mertzanidou, A., Smolders, I., Geens, M., Nguyen, H.T., Barbé, L., Sermon, K., and Spits, C. (2016). Higher-density culture in human embryonic stem cells results in DNA damage and genome instability. Stem Cell Rep. *6*, 330–341.

Lamm, N., Ben-David, U., Golan-Lev, T., Storchová, Z., Benvenisty, N., and Kerem, B. (2016). Genomic instability in human pluripotent stem cells arises from replicative stress and chromosome condensation defects. Cell Stem Cell *18*, 253–261.

Li, L., Wang, B.H., Wang, S., Moalim-Nour, L., Mohib, K., Lohnes, D., and Wang, L. (2010). Individual cell movement, asymmetric colony expansion, rho-associated kinase, and E-cadherin impact the clonogenicity of human embryonic stem cells. Biophys. J. *98*, 2442–2451.

Mantel, C., Guo, Y., Lee, M.R., Kim, M.K., Han, M.K., Shibayama, H., Fukuda, S., Yoder, M.C., Pelus, L.M., Kim, K.S., et al. (2007). Checkpoint-apoptosis uncoupling in human and mouse embryonic stem cells: a source of karyotpic instability. Blood *109*, 4518–4527.

Mayshar, Y., Ben-David, U., Lavon, N., Biancotti, J.C., Yakir, B., Clark, A.T., Plath, K., Lowry, W.E., and Benvenisty, N. (2010). Identification and classification of chromosomal aberrations in human induced pluripotent stem cells. Cell Stem Cell *7*, 521–531.

McClatchey, A.I., and Yap, A.S. (2012). Contact inhibition (of proliferation) redux. Curr. Opin. Cell Biol. *24*, 685–694.

Musacchio, A. (2015). The molecular biology of spindle assembly checkpoint signaling dynamics. Curr. Biol. *25*, R1002–R1018.

Nam, H.J., Naylor, R.M., and van Deursen, J.M. (2015). Centrosome dynamics as a source of chromosomal instability. Trends Cell Biol. *25*, 65–73.

Nguyen, H.T., Geens, M., Mertzanidou, A., Jacobs, K., Heirman, C., Breckpot, K., and Spits, C. (2014). Gain of 20q11.21 in human embryonic stem cells improves cell survival by increased expression of Bcl-xL. Mol. Hum. Reprod. *2*, 168–177.

Nichols, J., and Smith, A. (2012). Pluripotency in the embryo and in culture. Cold Spring Harb. Perspect. Biol. *4*, a008128.

Ohgushi, M., Matsumura, M., Eiraku, M., Murakami, K., Aramaki, T., Nishiyama, A., Muguruma, K., Nakano, T., Suga, H., Ueno, M., et al. (2010). Molecular pathway and cell state responsible for dissociation-induced apoptosis in human pluripotent stem cells. Cell Stem Cell *7*, 225–239.

Ohgushi, M., Minaguchi, M., and Sasai, Y. (2015). Rho-signaling-directed YAP/TAZ activity underlies the log-term survival and expansion of human embryonic stem cells. Cell Stem Cell *17*, 448–461.

Peterson, S.E., and Loring, J.F. (2014). Genomic instability in pluripotent stem cells: implications for clinical applications. J. Biol. Chem. *289*, 4578–4584.

Sakaue-Sawano, A., Kurokawa, H., Morimura, T., Hanyu, A., Hama, H., Osawa, H., Kashiwagi, S., Fukami, K., Miyata, T., Miyoshi, H., et al. (2008). Visualizing spatiotemporal dynamics of multicellular cell-cycle progression. Cell *132*, 487–498.

Santaguida, S., and Amon, A. (2015). Short- and long-term effects of chromosome mis-segregation and aneuploidy. Nat. Rev. Mol. Cell Biol. *16*, 473–485.

Spits, C., Mateizel, I., Geens, M., Mertzanidou, A., Staessen, C., Vandeskelde, Y., Van der Elst, J., Liebaers, I., and Sermon, K. (2008). Recurrent chromosomal abnormalities in human embryonic stem cells. Nat. Biotechnol. *26*, 1361–1363.

Suemori, H., Yasuchika, K., Hasegawa, K., Fujioka, T., Tsuneyoshi, N., and Nakatsuji, N. (2006). Efficient establishment of human embryonic stem cell lines and long-term maintenance with stable karyotype by enzymatic bulk passage. Biochem. Biophys. Res. Commun. *345*, 926–932.

Taapken, S.M., Nisler, B.S., Newton, M.A., Sampsell-Barron, T.L., Leonhard, K.A., McIntire, E.M., and Montgomery, K.D. (2011). Karotypic abnormalities in human induced pluripotent stem cells and embryonic stem cells. Nat. Biotechnol. *29*, 313–314.

Tanenbaum, M.E., and Medema, R.H. (2010). Mechanisms of centrosome separation and bipolar spindle assembly. Dev. Cell *19*, 797–806.

Watanabe, K., Ueno, M., Kamiya, D., Nishiyama, A., Matsumura, M., Wataya, T., Takahashi, J.B., Nishikawa, S., Nishikawa, S., Muguruma, K., et al. (2007). A ROCK inhibitor permits survival of dissociated human embryonic stem cells. Nat. Biotechnol. *25*, 681–686.

Whalley, H.J., Porter, A.P., Diamantopoulou, Z., White, G.R., Castañeda-Saucedo, E., and Malliri, A. (2015). Cdk1 phosphorylates the Rac activator Tiam1 to activate centrosomal Pak and promote mitotic spindle formation. Nat. Commun. *6*, 7437.

Woodcock, S.A., Rushton, H.J., Castañeda-Saucedo, E., Myant, K., White, G.R., Blyth, K., Sansom, O.J., and Malliri, A. (2010). Tiam1-Rac signaling counteracts Eg5 during bipolar spindle assembly to facilitate chromosome congression. Curr. Biol. *20*, 669–675.

Zhang, M., Cheng, L., Jia, Y., Liu, G., Li, C., Song, S., Bradley, A., and Huang, Y. (2016). Aneuploid embryonic stem cells exhibit impaired differentiation and increased neoplastic potential. EMBO J. *35*, 2285–2300.

Zhao, B., Zhang, W.D., Duan, Y.L., Lu, Y.Q., Cun, Y.X., Li, C.H., Guo, K., Nie, W.H., Li, L., Zhang, R., and Zheng, P. (2015). Filia is an ESC-specific regulator of DNA damage response and safeguards genomic stability. Cell Stem Cell *16*, 684–698.

Stem Cell Reports, Volume *9*

Supplemental Information

A RHO Small GTPase Regulator ABR Secures Mitotic Fidelity in Human Embryonic Stem Cells

Masatoshi Ohgushi, Maki Minaguchi, Mototsugu Eiraku, and Yoshiki Sasai

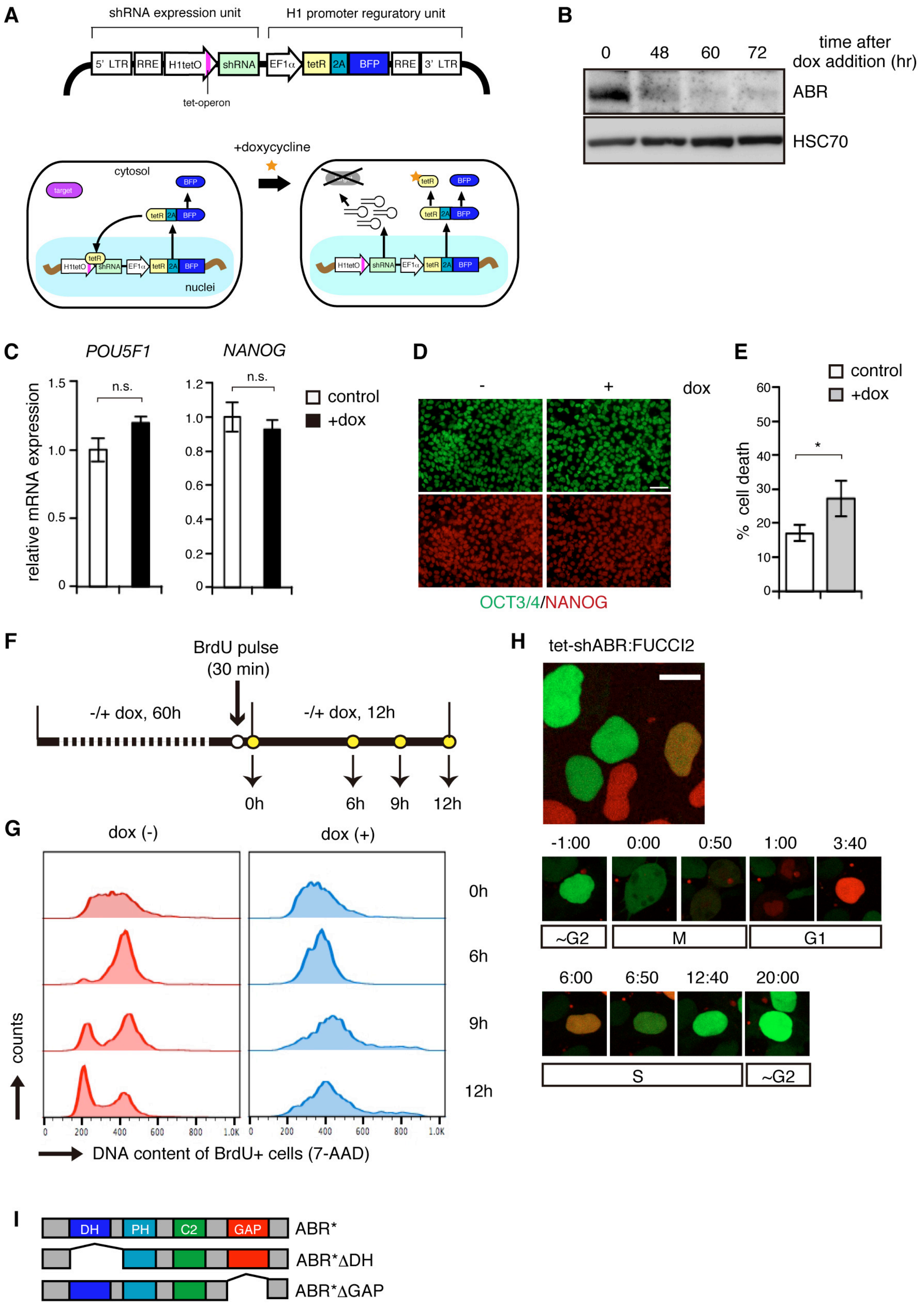
A
shRNA expression unit
H1 promoter reguratory unit
5' LTR
RRE
H1tetO
shRNA
EF1α
tetR
2A
BFP
RRE
3' LTR
tet-operon
+doxycycline
cytosol
target
nuclei
B
time after dox addition (hr)
0
48
60
72
ABR
HSC70
C
POU5F1
NANOG
relative mRNA expression
n.s.
control
+dox
D
dox
OCT3/4/NANOG
E
control
+dox
% cell death
*
F
BrdU pulse (30 min)
-/+ dox, 60h
-/+ dox, 12h
0h
6h
9h
12h
G
dox (-)
dox (+)
counts
DNA content of BrdU+ cells (7-AAD)
H
tet-shABR:FUCCI2
-1:00
0:00
0:50
1:00
3:40
~G2
M
G1
6:00
6:50
12:40
20:00
S
~G2
I
DH
PH
C2
GAP
ABR*
ABR*ΔDH
ABR*ΔGAP

Figure S1. ABR depletion strategy and phenotypic analyses, related to Figure 1.

(A) Overview of drug-inducible gene silencing strategy. The activity of a modified *H1* promoter was minimized by co-expressing Tet Repressor proteins (TetR) driven from a constitutively active *EF1A* promoter. Doxycyclin supplementation relieves the modified *H1* promoter from TetR, leading to the induction of shRNA transcription. LTR, long terminal repeat; RRE, rev response element; FP, fluorescence protein.

(B) Time course analysis of ABR protein downregulation.

(C-D) The effects of ABR depletion on pluripotent marker expression. Cells were cultured with or without dox for 3 days, and as markers for pluripotency, *POU5F1* and *NANOG* mRNA expression were evaluated by qPCR (C). Data are displayed as relative value to control. The protein abundance of OCT3/4 (green) and NANOG (red) were also evaluated by immunostaining (D). Scale bar represents 50 µm.

(E) Cell death assay. Tet-shABR KhES-1 cells were cultured with or without dox for 3 days. Dead cells were identified by an incorporation of live cell-impermeable DNA dye DRAQ7. DRAQ7-positive dead cells were determined by flow cytometric analyses.

(F-G) Tracing analyses of S-phase labeled cells. (F) A schematic diagram for the time schedule. S-phase cells were labeled by a transient supplementation of BrdU at 60 h time points during dox treatment. Then, cells were harvested at the indicated time point after BrdU washout, and subjected to staining and FACS analyses. (G) BrdU-positive cells were identified, and DNA contents of these cells were quantified by 7-AAD intensity. A peak with a low 7-AAD intensity represents the G1 population, and another peak with a high intensity represents G2-M populations.

(H) Live imaging analyses of FUCCI-expressing tet-shABR cells. Bottom pictures are snapshots from time-lapse tracing of single cells. t=0:00 corresponds to NEB. Scale bar represents 10 µm.

(I) Domain structure of ABR and its mutants.

The western blotting and BrdU assays were done two times (B and G). The immunostaining was repeated three times with three replicates in each experiment (D). The representative results were shown. Q-PCR experiments were repeated three times and data are shown as bar graphs (C and E). Error bars in graphs represent SD (C and E). Live imaging was performed as three independent experiments (H). Statistics: student's *t* test (C and E, n = 3); not significant (n. s.) and * $p < 0.05$.

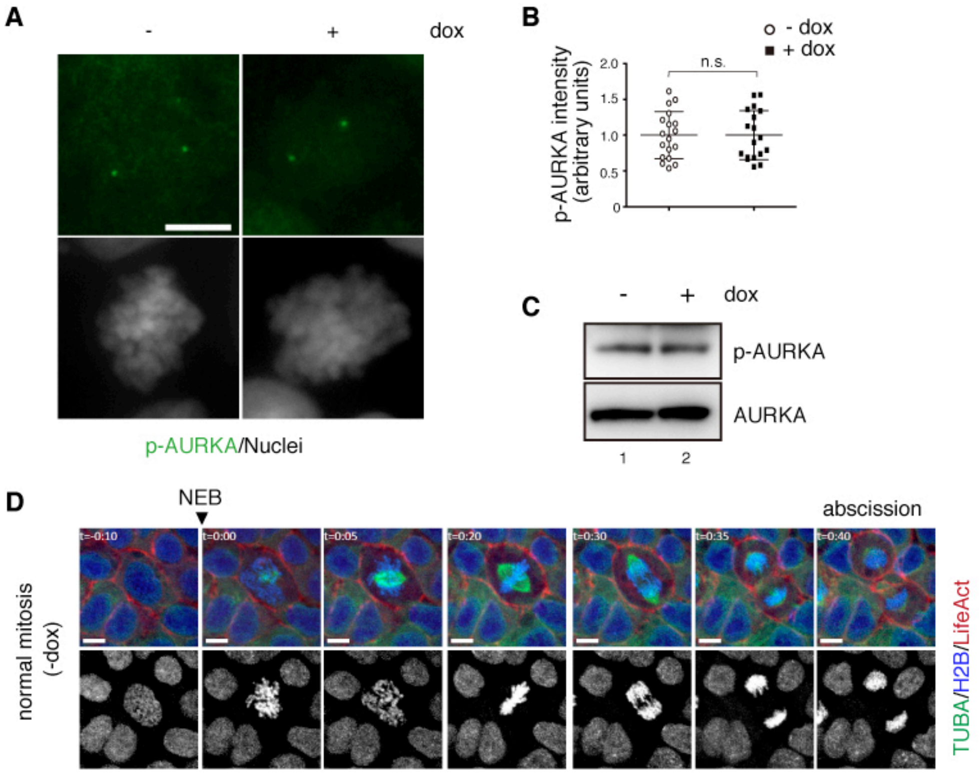

Figure S2. Abnormalities of ABR-depleted cells in centrosome separation and cell division, related to Figures 2 and 3.

(A-C) Evaluation of centrosome maturation. Cells were treated with or without dox for 3 days and phospholyration level of AURKA was examined by Immunostaining (A). Scale bar represents 10 μm. The signal intensity of paired centrosomal fluorescence was quantified by image analyses and shown as a scatter plot (B). Western blotting analyses were also done (C).

(D) A control experiment for live imaging of tet-shABR cells expressing fluorescent protein-tagged H2B (chromosome, bleu or gray), α-tubulin (TUBA, mitotic spindle, green), LifeAct (F-actin, red). t = 0 corresponds to NEB onset. Scale bars represent 10 μm.

The immunostaining was repeated three times with three replicates in each experiment (A and B). The western blotting was done three times (C). Error bars in graph represent SD (B). The imaging experiments were done three independent times. The representative examples were shown (A, C and D). Statistics: student's *t* test (B, n = 18 for control, n=18 for dox); not significant (n. s.).

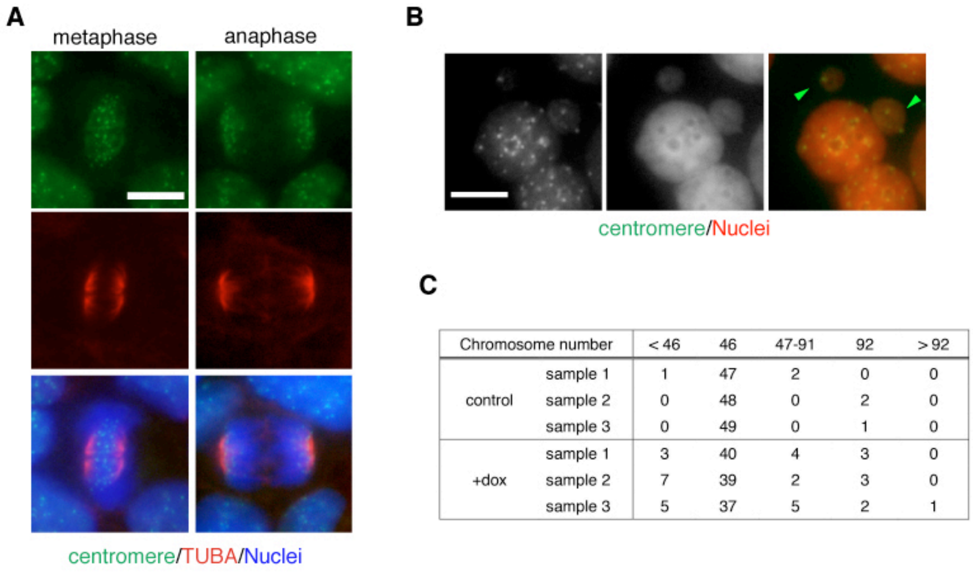

C

Chromosome number		< 46	46	47-91	92	> 92
control	sample 1	1	47	2	0	0
	sample 2	0	48	0	2	0
	sample 3	0	49	0	1	0
+dox	sample 1	3	40	4	3	0
	sample 2	7	39	2	3	0
	sample 3	5	37	5	2	1

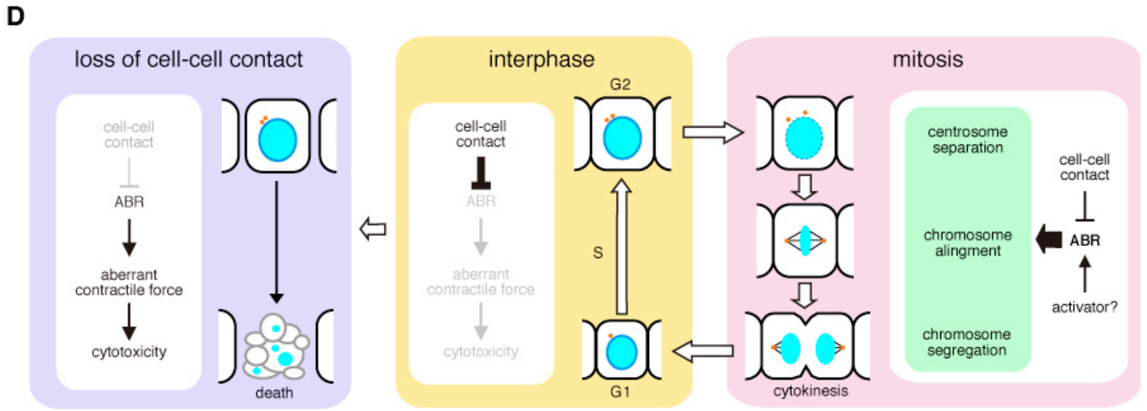

Figure S3. Chromosome segregation in ABR-depleted cells, related to Figure 4.

(A) Examples for a typical staining pattern of metaphase and anaphase cells. Centromere (green), TUBA (red), nuclei (blue). Scale bar represents 10 µm.

(B) Micronuclei in ABR-depleted cells (green arrowheads). Scale bar represents 10 µm.

(C) Summary of chromosome counting. Each mitotic spread was prepared from the cells that were treated with or without dox for 5 days, and subjected to DAPI staining. According to chromosome number, cells are categorized into the indicated five groups.

(D) Schematic diagram of ABR actions (see main text).

The immunostaining was repeated three times with five replicates in each experiment (A and B). The mitotic spreads for chromosome counting were prepared in three separated experiments (C).

Movie S1. Centrosome separation in ABR-depleted cells, related to Figure 2.

The tet-sABR hESCs expressing an mVenus-CENT2 were imaged for 12 hr. (part.1) control experiment. (part. 2) Time-lapse recording was performed in the presence of dox. t = 0 corresponds to the time point of separation initiation.

Movie S2. Mitotic progression in ABR-depleted cells, related to Figure 3.

The tet-sABR hESCs expressing a fluorescent protein-fused H2B (blue), TUBA (green), LifeActi (red) were imaged for 48 hr. (part. 1) control experiment. (part. 2~4) Time-lapse recording was performed in the presence of dox. Examples for cell death (part 2), cytokinesis failure (part 3) and extended mitosis (part. 4) are shown. t = 0 corresponds to NEB onset.

Movie S3. Chromosome segregation errors in ABR-depleted cells, related to Figure 4.

The tet-shABR hESCs expressing ECFP-fused H2B were imaged in the presence of dox. t = 0 corresponds to NEB onset.

SUPPLEMENTAL EXPERIMENTAL PROCEDURES

Cell culture.

All experiments using hESC lines were approved by an institutional ethics committee and done following the hES cell guidelines of the Japanese government. Undifferentiated hESCs were cultured on feeder layers of mouse embryonic fibroblasts (MEF; purchased from Kitayama Labes) in D-MEM/F12 (Sigma) supplemented with 20% KSR additive, 2 mM glutamine, 0.1 mM non-essential amino acids (Invitrogen), 5 ng/ml recombinant human bFGF (Wako) and 0.1 μM 2-ME under 2% CO_2. For cell passaging, hESC colonies were detached and recovered *en bloc* from the feeder layer by treating them with CTK dissociation solution at 37°C for 5-7 minutes, followed by tapping the cultures and flushing them with a pipette. The detached ESC clumps were broken into smaller pieces by gently pipetting them several times and then these small clumps were transferred onto a MEF-seeded dish. For feeder-free cultures, contaminating MEF cells were removed by incubating the cell suspension on a gelatin-coated plate at 37°C for 2 hours in the maintenance culture medium. The MEF-free hESCs were seeded and maintained on Matrigel substrate (BD Biosciences) in MEF-conditioned medium. The culture medium was refreshed daily until the next passage.

Plasmids and transfection

The cDNAs for *TUBA* and *CENT2* were amplified by PCR using PrimeSTAR GXL (Takara) using KhES-1 cDNA as a template. The generation of ABR mutants was described previously (Ohgushi et al, 2010). The LifeAct-TagRFP vector was purchased from Idibi. A lentivirus vector for FUCCI2 reporter was a gift from Dr. Miyawaki (RIKEN, BRC). The cDNAs for other fluorescence protein-fused proteins were obtained by PCR. All cDNAs were subcloned into the *pENTR/D* entry vector (Invitrogen) and subsequently sequenced. To generate stable cell lines, cDNAs were subcloned to piggybac transposon vectors containing a CAG-promoter driving expression cassette followed by an IRES-NeoR or an IRES-PuroR cassette.

The transfection to hESC with cDNA expression plasmids was performed with the FuGENE HD transfection reagent (Roche), as described previously (Ohgushi et al., 2010). To obtain stable transfectants, the cDNA expression cassettes were integrated into genomes using a piggybac transposon (PB) system. The PB vectors were co-transfected into hESC with a *pCAG-PBase* expression vector (Ohgushi et al., 2015). A few days after the transfection, cells were passaged to DR4 MEF (Cell Systems)-coated dishes and, on the following day, the medium was switched to a 100 μg/ml G418 or a 2 mg/ml puromycin-containing one. To avoid clone biases, we used the stable transfectants as a drug-resistant pool. In the case of introducing multiple transgenes, *PB-CAG-INeo* and *PB-CAG-IPuro* vectors were co-transfected and stable pools with both G418- and puromycin-resistance were selected.

For inducible ABR knockdown, we used the Tet-inducible shRNA expression lentivirus vector system. The preparation of lentivirus vectors, production of recombinant lentiviruses, infection to hESCs and FACS sorting were performed as previously described (Ohgushi et al., 2015). To induce shRNA expression, the culture medium was switched to a fresh one containing 1 μg/ml of dox, and the medium was changed daily until the analyses were completed. Note that, in the analyses of dox-treated samples,

dead or detached cells that emerged during the culture were excluded from the assay, because they were washed-out during the medium change.

Immunostaining, western blot analyses and quantitative real-time PCR

Immunostaining was performed as previously described (Watanabe et al., 2007). The cells were seeded onto a MEF-coated 8-well chamber slide, and fixed with 4% PFA at 4°C for 20 minutes and then permeabilized with 2% Triton-X100 solution. After incubation with blocking solution (2% skim milk), cells were incubated in the blocking solution containing specific antibodies. The staining was visualized using secondary antibodies conjugated with AlexaFluor-488, -546 or -647 (Invitrogen). Experiments were performed at least three times. Antibodies used in this work are listed below. For F-ACTIN staining, AlexaFluor-conjugated phalloidin (Invitrogen) was used. Nuclei were stained with DAPI or DRAQ5 (Cell Signaling). For analyses of metaphase-arrested cells, cells were treated with 1 μg/ml MG132 for 1 hr and then immediately subjected to immunostaining. Images were obtained with a fluorescence microscopy (AxioCam, Zeiss) or an inverted confocal microscopy (LSM780, Zeiss).

For the detection of endogenous protein expression in hESCs, cells were transferred onto Matrigel to minimize the possible contamination of MEF-expressing proteins into hESC lysates. After dox treatment, cells were washed with PBS, treated on the plate with HEPES lysis buffer (50 mM HEPES pH 7.4, 150 mM NaCl, 1 mM EDTA, 1 % NP-40 and protease inhibitor cocktail) for 10 min at 4°C with gentle shaking, and total cell extracts were harvested by pipetting. Immediately after adding the appropriate amount of 4 x SDS sample buffer to the extracts, they were subjected to a brief sonication for complete lysis. After boiling, the cell lysates were analyzed by SDS-PAGE and sequential western blot. A 5% skim milk solution was routinely used as a blocking reagent. Specifically, for the detection of phosphorylated proteins, 2% BSA solution was used for blocking. Images were obtained with a LAS3000 image analyzer (Fuji film).

Primary antibodies used in this work are listed below: anti-ABR (BD transduction, 611122), anti-ACTIN (Sigma, A5060), anti-p-AURKA (Cell Signaling, 3079), anti-centromere protein (Antibodies Incorporated, 15-235), HSC70 (Santa Cruz, sc-7298), anti-NANOG (R&D, AF1997), anti-OCT3/4 (BD Transduction, 611202), αTUBULIN (Millipore, MAB1864) and γTUBULIN (Sigma, T3559).

To evaluate mRNA expression, we performed quantitative PCR analyses. Total RNA was extracted using the RNAeasy Mini Kit (Quiagen) and then cDNAs were synthesized by SuperScript II reverse transcriptase (Invitrogen). The PCR reaction mixture was prepared on 96-well plate using a *Power* SYBR Green PCR Master Mix according to the manufacturer's instructions (Applied Biosystems). They were run in duplicate on a 7500 Fast Real-Time PCR System (Applied Biosystems). Expression level of each mRNA was estimated according to the corresponding standard curve and normalized to *GAPDH*. Data were displayed as arbitrary units or as relative values compared to each control. Primer sets used in this work are described in our previous paper (Ohgushi et al., 2015).

Cell cycle analyses.

To label replicating cells, cells were supplemented with BrdU and cultured for 40 min. After washing with PBS, the labeled cells were dissociated, harvested and stained using APC BrdU Flow Kit (BD

Pharmingen). The stained cells were analyzed by a Flow cytometer (BD Bioscience). To trace the cell cycle progression, the S-phase population was labeled by a transient supplementation of Brd-U. After complete washout of Brd-U, these cells were kept on culture to progress into the G2-M phase. Cells were harvested at 0, 6, 9 and 12 hours after labeling and DNA content was analyzed to trace the fate of Brd-U-positive cells. DNA contents were quantified by simultaneous staining with a 7-AAD DNA dye. Data were processed using FlowJo software (ver.12).

Karyotype analyses.

The dox-treated or -untreated cells were harvested after 2 hours treatment with 0.06 μg/ml Colcemid (Gibco). The cells were incubated in Buffered Hypotonic Solution (Genial Genetics) for 10 min, fixed by multiple changes of 3:1 methanol:acetic acid mixture, and then dropped onto dried glass slides. The condensed chromosomes are visualized by DAPI staining, and counted under the microscope. Since chromosome identification was relatively difficult in the case of dox-treated samples due to contamination of dead cell-derived DNA debris, one control and two dox-treated samples were analyzed by a professional (Chromosome Science Lab) and confirmed to obtain the identical results. To identify each chromosome, the slides were analyzed by multi-color fluorescence in situ hybridization (mFISH) using a 24XCyte Multi Color Probe Kit (MetaSystems). Probe hybridization was done using a VP2000 Processor (Abbott). Images were obtained with an MSearch imaging system and processed with ISIS software (MetaSystems).

Live imaging.

For live imaging, hESC clumps were seeded onto a MEF-coated 35-mm μ-dish (Ibidi), and they were imaged on an inverted microscope (IX81-ZDC, Olympus) that was equipped with a stepper filter wheel (Ludl) and a cooled EM-CCD camera (ImagEM, Hamamatsu Photonics). For confocal observations, serial images were collected using a CSU-W1 unit (Yokogawa) configured with an IX81-ZDC microscope. To observe centrosome behaviors, 8 images were obtained with 0.25 μm intervals along the Z-plane. Time-lapse recording was started after the 60 h dox treatment and was done for the following 12 h with 2-min time intervals. In the case of the observation of mitotic progression, 10 images were obtained with 1-μm intervals along the Z-plane. After 24 h dox treatment, recording was done for the following 46 h with 5-min time intervals. In both cases, NEB is identified as a shift of non-centrosomal fluorescence signal from cytosolic to diffuse pattern. The maximum projection image was constructed from the obtained slices using MetaMorph software.

Statistical analyses.

Error bars in the figures represent standard deviations. Statistical significance was tested by Student's t-test for two-group comparison, and by one-way ANOVA for multi-group comparison with Dunnett's test using Prism4 software (GraphPad).

SUPPLEMENTAL REFERENCES

Ohgushi, M., Minaguchi, M. and Sasai, Y. (2015). Rho-signaling-directed YAP/TAZ activity underlies the log-term survival and expansion of human embryonic stem cells. Cell Stem Cell *17*, 448-461.

Ohgushi, M., Minaguchi, M. and Sasai, Y. (2015). Rho-signaling-directed YAP/TAZ activity underlies the log-term survival and expansion of human embryonic stem cells. Cell Stem Cell *17*, 448-461.

Watanabe, K., Ueno, M., Kamiya, D., Nishiyama, A., Matsumura, M., Wataya, T., Takahashi, J.B., Nishikawa, S., Nishikawa, S., Muguruma K., et al. (2007). A ROCK inhibitor permits survival of dissociated human embryonic stem cells. Nat. Biotechnol. *25*, 681-686.
